# Multi-level transcriptional control of specific macrophage responses

**DOI:** 10.1038/s44318-026-00898-7

**Published:** 2026-08-13

**Authors:** Alexandra Drakaki, Josje M A Huisman, Marten A Hoeksema

**Affiliations:** Department of Medical Biochemistry, Amsterdam Institute for Immunology and Infectious Diseases; Amsterdam Cardiovascular Sciences Institute, Amsterdam UMC, Location AMC, Amsterdam, The Netherlands

**Keywords:** Chromatin, Transcription & Genomics, Immunology

## Abstract

Macrophages can adopt diverse functional states in response to environmental cues, a process that is fundamentally controlled at the level of transcriptional regulation. In this review, we outline a hierarchical framework of transcription factor activity that underpins macrophage identity and activation. Firstly, lineage-determining transcription factors establish the cell-type-specific chromatin landscape during development. Upon tissue seeding, local signals shape the activity of additional transcription factors that refine enhancer landscapes and drive tissue-specific macrophage phenotypes. Macrophages further adopt their functional states when exposed to potential threats to tissue homeostasis, such as bacterial ligands or inflammatory cytokines. In response, signal-dependent transcription factors are activated and initiate signal-appropriate transcriptional programs. The specificity and durability of these responses are determined by secondary transcription factors that modulate the magnitude, timing, and maintenance of stimulus-induced transcriptional programs. Collectively, macrophage responses emerge from the activity of interconnected layers of cell-type-, tissue-, and signal-dependent transcription factors, forming complex regulatory networks that inflict stimulus-specific outcomes. Dysregulation of these networks can transcriptionally rewire macrophages toward disease-associated states. Delineating transcriptional regulators that distinguish signal responses may enable more targeted disease interventions.

## Abstract (web summary)

This review discusses how different layers of transcription factor activity cooperate to yield stimulus-specific macrophage responses.

## Graphical abstract

**Figure:**
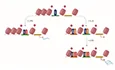

## Introduction

In order to orchestrate a timely response against potential threats, immune cells need to coordinate their activities. Signaling mediators, including cytokines, growth factors, and lipids, serve as the immune system’s communication mediators that instruct appropriate cell responses (Lin and Leonard, 2019). The effects elicited by the signaling molecules, however, are highly context-dependent and depend on cell type and tissue context. Single-cell transcriptomic profiling of immune cell responses to each of 86 cytokines in mouse lymph nodes revealed the induction of highly cell-type-specific gene programs (Cui et al, 2024). Cells that originate from the same lineage but populate distinct tissues frequently adopt divergent transcriptional and functional states in response to environmental cues, with tissue-resident macrophages (TRMs) being a characteristic example (Lazarov et al, 2023; T’Jonck et al, 2018).

Macrophages are widely considered the sentinel cells of the immune system. Depending on origin and tissue of residence, they adopt specialized roles that extend beyond their shared effector functions (Mass et al, 2023). Advancements in lineage tracing and fate-mapping models, in combination with the rise of omics technologies, have provided major insights into the mechanisms that govern macrophage identity and activation. Following years of scientific debate, it is now widely accepted that most resident macrophage subsets originate from embryonic precursors that seed tissues before birth and retain proliferative capacity throughout life. Macrophages in adult tissues can also differentiate from an alternative lineage represented by bone marrow-derived monocytes. Monocyte-derived macrophages (MDMs) can either adopt an identity that is nearly identical to their embryonic-derived TRM counterparts or, responding to tissue injury, take on non-homeostatic functions associated with the coordinated response to damage. Macrophages retain a remarkable level of plasticity after differentiation, allowing them to swiftly adapt their functional states in response to cues present in their microenvironment. As such, macrophages provide a powerful model to study how extracellular signals are translated into context-specific gene regulatory programs.

Macrophage responses to signal encounters are encoded at the level of transcriptional and epigenetic regulation, which is shaped by developmental origin and tissue imprinting (Chen et al, 2020; Lazarov et al, 2023). The process of establishing macrophage chromatin states is carried out by a hierarchical network of transcription factors (TFs) that gain control in a successive manner. Firstly, lineage-determining transcription factors (LDTFs) become activated in macrophage precursors and play a central role in opening up the chromatin at thousands of promoters and enhancers that will dictate a macrophage-specific identity (Box 1). Upon tissue colonization, local signals imprint context-specific functions and diversify the transcriptional programs of incoming cells by means of selective expression of an additional set of TFs that are unique to each tissue, referred to as tissue-restricted transcription factors (TRTFs). Macrophages are continuously exposed to inflammatory or (non-)homeostatic cues, requiring ongoing adaptability in their gene expression programs. These responses are influenced by the pre-configured enhancer landscape set in place by constitutively active LDTFs and context-dependent TFs, while signal-induced gene expression is initiated rapidly by primary signal-dependent TFs (SDTFs). The specificity, magnitude, and persistence of these responses are subsequently refined by additional transcriptional regulators or so-called secondary SDTFs induced in a context-dependent manner (Huisman et al, 2026).

In this review, we discuss the hierarchical transcriptional networks that establish (tissue-resident) macrophage identity and signal-dependent transcriptional regulation in macrophages. Given the breadth of macrophage biology, we focus specifically on TF-mediated regulation of macrophage identity and activation. We do not aim to cover other regulatory mechanisms, including non-coding RNAs, post-transcriptional or metabolic control. Here, we propose a conceptual framework in which signal-dependent transcriptional regulation in macrophages is organized into two distinct layers. The first drives a rapid and relatively broad gene expression program, while the second encodes response specificity through stimulus-dependent chromatin remodeling and the establishment of transcriptional memory. We argue that this second layer is fundamentally shaped by the epigenetic landscape established during ontogeny and tissue imprinting—such that differences in chromatin accessibility between a Kupffer cell, an alveolar macrophage, and an inflammation-associated MDM will produce divergent transcriptional responses to the same stimulus. Macrophages are continuously exposed to numerous and often antagonistic signals in their environment. The response to this layered and conflicting input is not a sequential adaptation to the latest signal, but rather a continuous integration of signal information. We describe how secondary SDTFs integrate this information in a context-dependent manner through cooperative or competitive interactions. Lastly, we examine how aberrant signaling networks in disease rewire the macrophage enhancer landscapes that contribute to pathogenic activation states. Together, this review provides a conceptual framework for understanding how macrophages integrate developmental, tissue-derived and environmental signals into context-specific transcriptional programs in health and disease.

Box 1 Enhancers and LDTFs Precise control of gene expression in macrophages relies on coordinated communication between gene promoters and distal regulatory enhancers. Promoters, which define transcription start sites, are typically marked by H3K4me3, whereas enhancers are characterized by H3K4me1 and reside at variable distances from their target genes (Heintzman et al, 2007). Although both regulatory elements harbor TF-binding motifs and acquire H3K27 acetylation upon activation, enhancers exhibit cell-type specificity, forming the principal basis of macrophage-specific gene regulation (Romanoski et al, 2015).

This specificity is established by lineage-determining transcription factors (LDTFs), including PU.1, AP-1, and C/EBPβ, which play central roles in macrophage differentiation and identity (Heinz et al, 2010). These factors often act as pioneer factors, accessing their binding motifs within closed chromatin and cooperating with one another to select and establish macrophage-specific enhancers (Glass and Natoli, 2016; Gosselin et al, 2014a). LDTF binding promotes recruitment of chromatin remodeling complexes, resulting in local chromatin opening and deposition of H3K4me1, thereby generating a primed enhancer state (Ghisletti et al, 2010). These primed enhancers can engage in or stabilize enhancer–promoter looping interactions, positioning them to regulate transcription upon stimulation.

## Transcription factor specification in response to tissue-derived signals

Fate-mapping and lineage tracing studies in mice have highlighted that adult tissue macrophages mainly originate from two types of progenitors. TRMs mainly derive from erythro-myeloid progenitors (EMPs) of the yolk sac, while a few populations, in both homeostatic and non-homeostatic conditions, can be differentiated from hematopoietic stem cells (HSCs) (Ginhoux et al, 2010; Gomez Perdiguero et al, 2015; Guilliams et al, 2013; Hashimoto et al, 2013; Hassnain Waqas et al, 2017; Schulz et al, 2012). EMPs arise from the yolk sac during the second wave of hematopoiesis at embryonic day 8.25 (E8.25) and later migrate into the fetal liver. A fraction of these cells differentiates into EMP-derived macrophage precursors (preMacs), which go on to colonize different embryonic tissues, starting at E9.5 (Lazarov et al, 2023). This event coincides with the onset of organogenesis. HSCs, instead, emerge from the aorto-gonado-mesonephros (AGM) region and also migrate to the fetal liver, where they expand in numbers. HSCs finally colonize the bone marrow, where active hematopoiesis takes place, giving rise to cells of the erythroid, lymphoid, and myeloid lineages throughout adulthood (Perdiguero and Geissmann, 2016). HSC-derived macrophage input in adult tissues increases with age and differs significantly among organs, with minimal contribution observed in the brain, for example, while barrier tissues, including the dermis and the gut lamina propria, require constant replenishment (Bain et al, 2014; Tamoutounour et al, 2013). In addition, in the presence of tissue damage or infection, HSC-derived circulating monocytes home to the site of injury and differentiate locally into macrophage subsets that contribute to the inflammatory response, as well as its resolution (Yona et al, 2013).

**Figure 1:**
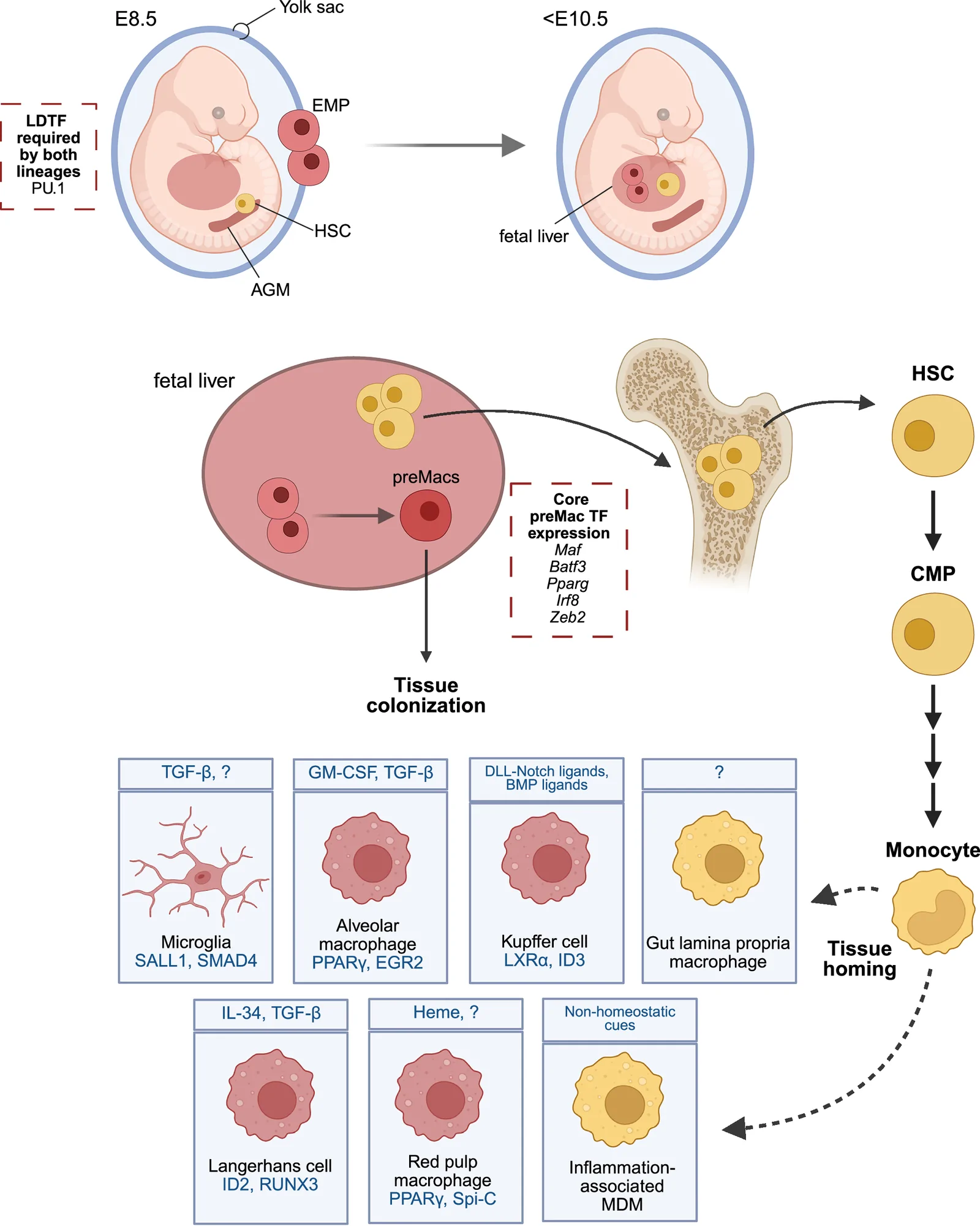
Transcription factor activation during macrophage development. LDTFs remodel chromatin and prime enhancers that establish a macrophage-specific identity. The LDTF PU.1 is required in macrophage progenitors of both EMP and HSC lineages prior to fetal liver seeding. In the fetal liver, EMP-derived preMacs acquire a core macrophage program, including TFs *Maf, Batf3, Pparg, Irf8*, and *Zeb2*, before colonizing tissues where local cues drive tissue-specific TF expression and niche-restricted gene programs. HSC-derived cells instead colonize the bone marrow, retain proliferative capacity, and generate multiple lineages throughout adulthood, including monocytes, which can home into tissues during inflammation to differentiate into macrophages or contribute to the TRM pool. LDTF lineage-determining transcription factor, HSC Hematopoietic stem cell, EMP erythro-myeloid progenitor, AGM aorta-gonad-mesonephros, preMac EMP-derived macrophage precursor, CMP common myeloid progenitor, MDM monocyte-derived macrophage. Illustrations were created with BioRender.

In addition to the main functions that characterize macrophage biology, resident macrophages display context-specific functions to meet the requirements dictated by their niche (Mass et al, 2023). The functional heterogeneity of TRMs is largely encoded at the transcriptional level and it is achieved by the collaborative action of gradually diverging sets of TFs that guide macrophage precursors along the differentiation and specification trajectory. First, cells of the EMP and HSC lineages require the expression of a certain set of lineage-determining TFs before seeding the fetal liver. LDTFs can bind and open up the chromatin at *cis*-regulatory elements (CREs), establishing a chromatin context that will dictate a cell-type-specific identity. A characteristic example of an LDTF that controls macrophage development is the Ets-domain TF PU.1, whose absence renders mice deficient in TRMs (DeKoter and Singh, 2000; Scott et al, 1994). In the fetal liver, EMP-derived preMacs acquire a core macrophage transcriptional program that includes the TFs *Maf*, *Batf3*, *Pparg*, *Irf8*, and *Zeb2*, suggesting their involvement in imprinting a macrophage-specific identity (Mass et al, 2016; Scott et al, 2018). After macrophage LDTFs establish the cell-type-specific CRE landscape, preMacs begin to exhibit transcriptional heterogeneity upon colonization of the different embryonic tissues, which is achieved and maintained in an additional layer of transcriptional regulation by tissue-restricted transcription factors (TRTFs) (Mass et al, 2016) (Fig. 1).

Development studies have highlighted many of these TRTFs and the mechanisms behind their tissue-specific selection. Given the existing literature, two models of tissue specification can be rationalized. The first one suggests that a polarizing signal, such as a cytokine or growth factor, or simple combinations of those that predominate in a specific milieu, instructs the preferential expression of tissue-specific transcriptional regulators. Alternatively, individual preMacs that reach the different embryonic tissues stochastically express distinct combinations of TRTFs. The cells marked by the TFs whose expression is reinforced by the specific environmental signals have a survival advantage and outgrow the competing clones. This eventually leads to the establishment of a tissular niche that not only ensures macrophage self-maintenance but also confers a tissue-specific identity (Lazarov et al, 2023). Regardless of the exact process governing macrophage specification, it is clear that niche-restricted cues in vivo are essential for imprinting the tissue-specific identity of macrophages through the induction of key TFs (Fig. 1; Table 1). Below, we discuss some key examples of TRTFs and the environmental cues that control their activation.

**Table 1:** Environment signals and transcription factors controlling tissue-resident macrophage identity.

Tissue-resident macrophage		Organ	Tissue signal(s)	TF(s) specifying identity during development	
Alveolar macrophage		Lung	GM-CSF, TGF-β	PPARγ, EGR2	
Lung interstitial macrophage		Lung interstitium	TGF-β, CSF1	Mafb (Nerve-associated)	Mafb, c-Maf (Perivascular)
Nerve-associated	Perivascular				
Red pulp macrophage		Spleen	Heme?	PPARγ, Spi-C	
Langerhans cells		Skin	IL-34, TGF-β	Runx3, Id2	
Kupffer cells		Liver	DLL-Notch and BMP ligands	LXRα, ID3, Smad4	
Microglia		Brain	TGF-β?	Sall1, Smad4	

### Lung macrophages

#### Alveolar macrophages

Alveolar macrophages (AMs) constitute the predominant macrophage subset residing in the lung, whose identity is largely controlled by PPARγ and partially by EGR2. The expression of both TFs is controlled by GM-CSF and TGF-β signaling. GM-CSF was initially described as a soluble mediator secreted by epithelial cells and fetal macrophages that is able to induce PPARγ in AMs (Guilliams et al, 2013; Schneider et al, 2014). Later, embryonic development of AMs and maintenance in adult tissues was also shown to be dependent on autocrine TGF-β signaling, which converges with GM-CSFR signaling in inducing *Pparg* expression (Yu et al, 2017). Despite being considered the “master TF” of AMs, conferring AM signature gene expression, involving lipid transport and metabolic pathways, PPARγ induces a distinct gene expression program in other macrophage subsets, including splenic red pulp macrophages (RPMs) (Okreglicka et al, 2021). Motivated by this discrepancy, McCowan et al explored the possibility that additional TFs are required to convey specificity during AM differentiation (McCowan et al, 2021). The authors demonstrated that EGR2, although not required for their development and survival, is indispensable for imprinting key phenotypic features in AMs. For example, alveolar macrophages of mice with myeloid lineage-specific *Egr2* deficiency displayed defective handling of bacteria when exposed to *Streptococcus pneumoniae*. This phenotype could be attributed to the reduced expression of genes encoding for molecules involved in bacterial recognition, capture, and killing when compared to their *Egr2*-sufficient counterparts. In addition, reconstitution of the depleted alveolar macrophage compartment following bleomycin-induced lung injury was considerably impeded in *Egr2*-deficient mice, where recruited monocytes fail to properly differentiate for effective tissue repair. Mechanistically, EGR2 expression is first detectable in neonatal AMs and maintained in adulthood, and is driven by TGF-β and GM-CSF signaling in a PPARγ-dependent manner. Notably, EGR2 was also shown to modulate the expression of C/EBPβ, another TF implicated in the control of alveolar macrophage differentiation (Dörr et al, 2022).

#### Interstitial macrophages

The interstitial space that provides structure and support to the lungs is also home to different macrophage subsets, specifically blood vessel-associated, or perivascular, CD206^+^ interstitial macrophages (IMs), and nerve-associated CD206^−^ IMs. Both of these subsets seem to be dependent on endothelial cell-derived TGF-β-activated MafB (Peng et al, 2025). Specifically, using an IM niche depletion model, Vanneste and colleagues showed that infiltrating monocytes initially restrict TF MafB expression to facilitate cell proliferation (Vanneste et al, 2023). Following this initial proliferative stage, transitioning monocytes require MafB to halt proliferation and “commit” to differentiation, before branching into either of the two defining subsets. Specifically, mice with a *Mafb* deletion lack lung IMs and gain a distinctive cell population that resembles transitioning monocytes, indicating that these cells are arrested in a pre-macrophage state. Mechanistically, monocytes infiltrating the interstitium encounter the CSF1 ligand, which leads to the upregulation of TGFβRII expression and TGF-β responsiveness. Then, endothelial cell-derived TGF-β1 potentiates MafB expression at both transcript and protein levels, subsequently activating MafB-dependent pathways and promoting IM differentiation (Peng et al, 2025). The myeloid-restricted TF c-Maf has also been explored for its contribution to the specification of IM subsets. Although not required for IM development, c-Maf is important for defining perivascular IM identity, specifically highlighted by the decrease in *Folr2* gene and CD206 protein expression in c-Maf-deficient IMs (Vanneste et al, 2023). The dependency of perivascular macrophages on c-Maf is not limited to the lung, as it has also been identified as a central regulator of the transcriptional program of vasculature-associated macrophages in the brain, intestine, and white adipose tissue (Moura Silva et al, 2021). This points to a shared mechanism of TF specification, possibly related to signals derived from the cells that make up the vasculature.

### Splenic macrophages

The normal development and maturation of splenic iron-recycling macrophages, or red pulp macrophages (RPMs), is significantly perturbed in the absence of PPARγ and heme-induced Spi-C (Okreglicka et al, 2021). Following colonization of fetal macrophage precursors in the spleen, PPARγ mediates the expression of genes related to cell adhesion, possibly allowing sufficient seeding of progenitors and differentiation into mature RPMs. The TF Spi-C is required for the maintenance and identity of the RPM pool in adulthood, potentially through its influence on PPARγ expression (Haldar et al, 2014; Kohyama et al, 2009; Okreglicka et al, 2021). The environmental cues steering TF activation in this setting are not fully elucidated, but IL-4 and GM-CSF seem to be dispensable for this (Okreglicka et al, 2021). One very important niche signal that reinforces RPM identity, however, comes from their primary function of recycling iron from senescent red blood cells. Specifically, RPMs degrade hemoglobin and produce the intermediate molecule heme. Heme can bind the negative regulator of Spi-C, Bach1, and mark it for proteasomal degradation, thereby allowing for Spi-C activation in these cells (Haldar et al, 2014). The literature discussed so far suggests that distinct tissue signals can converge on activating the same “master” TF (e.g., PPARγ in both RPMs and AMs), which may act cooperatively with additional TFs or specify downstream transcriptional regulators (Spi-C or EGR2, respectively). This tissue-specific TF network inflicts distinguishable gene programs.

### Langerhans cells

Langerhans cells (LCs) of the epidermis require both the alternative CSF1R ligand, IL-34, produced by local keratinocytes, and TGF-β1, produced both by keratinocytes and LCs themselves, and the TFs Id2 and Runx3 for normal development and tissue-specific specialization (Borkowski et al, 1996; Wang et al, 2012). TGF-β1 signaling promotes the expression of *Id2* and *Runx3*, individual deletion of which abrogates LC development (Fainaru et al, 2004; Hacker et al, 2003). Runx3 does not act as an upstream regulator of Id2, suggesting that these TFs have discrete, non-overlapping functions; however, the inverse relationship has not been determined to our knowledge (Fainaru et al, 2004).

### Liver Kupffer cells

In the liver, Kupffer cell identity is endowed by DLL-Notch-activated LXRα, Id3, and BMP ligand-activated Smad4. Studies employing KC depletion models have demonstrated that recruited monocytes quickly gain a KC-resembling gene expression program upon entering the hepatic niche (Bonnardel et al, 2019; Sakai et al, 2019). In the liver, KCs not only exist in the lining of the blood vessels, but also occupy the perisinusoidal space, where they closely interact with hepatic stellate cells, liver sinusoidal endothelial cells (LSECs), and hepatocytes. The precise engraftment of monocytes in this system is crucial for receiving all the necessary signals to acquire KC identity. Specifically, DLL-Notch ligands derived from LSECs promote the expression of transcriptional regulators that control KC identity, including LXRα and Id3. This TF network activates enhancers that support KC differentiation. Accessibility of hundreds of KC-specific enhancers is lost upon KC-specific deletion of LXRα, while embryonic loss of *Id3* confers liver macrophage deficiency (Mass et al, 2016; Sakai et al, 2019). In addition, TGF-β family BMP ligands from hepatic stellate cells and LSECs, and desmosterol produced in the hepatic microenvironment, further activate SMAD and LXR TFs to sustain KC identity. The TGF-β superfamily receptor Alk1, in particular, and its ligands, BMP9 and BMP10, instruct KC signature gene expression through Smad4 activation. Inhibition of this signaling pathway leads to perturbations in characteristic KC morphology and localization, and increases susceptibility to invading pathogens (Zhao et al, 2022).

### Microglia

The most characteristic example of regulatory element-mediated imprinting of macrophage identity comes from microglia, the main TRM population of the central nervous system (CNS) (Gomez-Nicola and Perry, 2014). The expression of *Sall1*, encoding a transcriptional regulator, is highly specific to microglia, and not other TRMs or CNS-resident cells (Fixsen et al, 2023). An enhancer knockout model has revealed that expression of *Sall1* is regulated by a microglia-specific super enhancer (SE) in a SMAD4-dependent manner. Pervasive TGF-β signaling in the brain activates the latter TF, which is further directed by Sall1 to bind key regulatory elements that imprint the identity and function of CNS-resident macrophages (Fixsen et al, 2023). Interestingly, BMDMs that infiltrate the brain upon microglia depletion can acquire part of the microglia gene signature and phenotypic attributes, but are unable to induce *Sall1* and lack a major part of the microglia-specific identity even months after seeding. Instead, they display a more “activated” inflammatory profile (Buttgereit et al, 2016). Given that engrafted BM-derived cells are also exposed to TGF-β, activation of SMAD4 should not be the limiting factor; instead, it is possible that the SE controlling *Sall1* is not accessible in BMDMs. In line with this, Lavin et al showed that microglia have one of the lowest percentages of shared enhancers with monocytes among several TRM subsets (Lavin et al, 2014). This discrepancy could be attributed to the distinct ontogeny of EMP-derived and HSC-derived macrophage populations.

### Macrophage plasticity

Environment cues are, in most cases, sufficient to induce TRTFs and drive macrophage specification, which has been highlighted by models of TRM depletion, whereby recruited monocytes are largely capable of differentiating into self-renewing resident macrophages (Bonnardel et al, 2019; Borkowski et al, 1996; Fainaru et al, 2004; Fixsen et al, 2023; Gomez-Nicola and Perry, 2014; Hacker et al, 2003; Kohyama et al, 2009; Okreglicka et al, 2021; Peng et al, 2025; Sakai et al, 2019; Schneider et al, 2014; Vanneste et al, 2023; Wang et al, 2012; Yu et al, 2017). The extent of macrophage plasticity, however, has been a topic of debate. Gosselin and colleagues reported that depriving TRMs from their native tissue context results in a loss of their tissue-specific gene programs, even in the presence of factors that support their survival and differentiation in vivo, including macrophage colony-stimulation factor (M-CSF) and IL-34. However, supplementation with tissue-specific cues induced epigenetic and transcriptional rewiring, driving a substantial shift toward the identity of the TRM normally residing in the corresponding tissue (Gosselin et al, 2014b). Similarly, Lavin and colleagues used ectopic transfer experiments and argued that mature peritoneal macrophages retain the capacity to be reprogrammed, adapting their transcriptomes to more closely resemble the identity of AMs when introduced into the respective tissue niche (Lavin et al, 2014).

Although Lavin et al suggested that even fully differentiated TRMs display extensive plasticity, at least at the transcriptomic level, recovery of transferred cells two weeks after being introduced to an intact AM niche was notably low, possibly explaining the lack of epigenetic profiling of these cells (Lavin et al, 2014). In addition, the ability of transferred cells to assume AM functions or to significantly contribute to the AM pool was not assessed. A subsequent report questioned the capacity of fully differentiated macrophages to phenotypically switch. Specifically, the authors demonstrated that macrophages isolated from the liver, colon, and peritoneum could not efficiently colonize the empty AM niche of *Csf2rb*^*−*/−^ mice, or perform essential AM functions, as evidenced by defects in surfactant clearance. However, yolk sac- and fetal liver-derived precursors, as well as adult bone marrow monocytes, were all capable of occupying the empty niche and generating AMs (van de Laar et al, 2016).

The displacement of resident macrophages from their niche likely removes the layer of regulation implemented by TRTFs, the expression of which is maintained by tissue signals. This leads to a disproportionate loss of macrophage subset-specific enhancers, some of which can be retrieved after experimental supplementation of the primary signaling factor present in the original environment (Gosselin et al, 2014b). Nevertheless, activation of some enhancers cannot be replicated in vitro, suggesting that other parameters, like additional cues, cell-cell interactions, and ontogeny, come into play that further fine-tune the epigenetic state of TRMs. It is important to note, however, that removal of a resident macrophage from its tissue niche does not result in replacement with a precursor-like cell with the plasticity characteristic of yolk sac- and fetal liver-derived precursors or bone marrow-derived monocytes. Instead, the plasticity of mature resident macrophages seems to be more limited, suggesting that some of the epigenetic rewiring associated with tissue imprinting is permanent. This is supported by studies of tissue perturbation, whereby the inflammatory response is mainly driven by recruited monocyte-derived macrophages that adopt transcriptional states markedly distinct from those of resident cells, which largely remain transcriptionally stable (Dick et al, 2019; Yamasaki et al, 2014; Zigmond et al, 2014). The recent study of Lohrmann et al further highlights this, as *Irf8* knockout mice with monocytopenia are capable of developing the full dermal macrophage subset diversity under steady state conditions but fail to mount a proper antimycobacterial response that requires monocytic influx (Lohrmann et al, 2025).

MDMs recruited to sites of inflammation are later shaped by local cues to more closely resemble a TRM identity, after the initial tissue threat is restrained (Blériot et al, 2020). Thereby, adult tissues are represented by a mosaic of TRMs of embryonic and bone marrow origin. These cells retain a memory of the reason they were recruited in a tissue at the epigenetic level. Despite not mounting a specific response, similarly to lymphocytes, macrophages respond to future challenges in a manner that is informed by the immune history of the niche they reside in (Netea and Joosten, 2024; Netea et al, 2016).

Taken together, these observations recapitulate a model of macrophage specification, whereby tissue signals inform the differentiation pathways that cells undergo. Embryonic precursor cells and bone marrow-derived monocytes are extensively malleable, adopting the specific chromatin landscapes, transcriptional signatures, and functional properties dictated by the environmental cues provided by the niche in which they engraft. This process is carried out by the activity of distinct sets of transcriptional regulators that act alone and in collaboration with ubiquitously expressed LDTFs (Gosselin et al, 2014b; Lavin et al, 2014). TRM maturation seems to involve some permanent epigenetic rewiring, highlighted by the inability of these cells to efficiently adopt the identity of other macrophage subsets upon tissue transfer (van de Laar et al, 2016). Consistent with their remarkable ability to assume virtually any TRM identity upon engraftment, monocyte-derived macrophages that infiltrate tissues following injury can similarly adopt a wide range of functional profiles, depending on local disease-associated signals and the stage of the inflammatory response. TRMs, on the other hand, prioritize repopulating their niche following tissue perturbation, while maintaining an overall unperturbed profile, implying tight regulation of the mechanisms that preserve TRM identity (Dick et al, 2019; Yamasaki et al, 2014; Zigmond et al, 2014). The signaling cascades that govern homeostasis in macrophages residing in adult tissues have not been thoroughly described; however, the JAK–STAT pathway was recently shown to play a prominent role (Fortelny et al, 2024) (Box 2).

Box 2 JAK–STAT signaling-mediated maintenance of tissue macrophage homeostasis As an integral part of innate immunity, macrophages constitute one of the first responders to implicit threats, including infection or tissue damage. Consequently, “resting” macrophages still need to be in a state of alertness in order to swiftly respond to danger cues. Recently, the JAK–STAT pathway was proposed to sustain a poised epigenetic and transcriptional state under homeostatic conditions that supports a swift immune response (Fortelny et al, 2024). Integrative transcriptomic analysis of macrophages isolated from the spleen of different JAK–STAT mutant mice revealed shared and distinct gene modules regulated by the constituents of this pathway. For example, ablation of members of the ISGF3 complex, namely STAT1, STAT2, and IRF9, showed downregulation of interferon-stimulated gene (ISG) expression, suggestive of low-level, constitutive interferon (IFN) signaling in homeostatic macrophages. Deprivation of wild-type macrophages from their in vivo context leads to transcriptional reprogramming resembling that of mutant cells, suggesting that tissue signals support this baseline signaling. Interestingly, there was not a single gene signature shared across all mutants, emphasizing the divergent and non-redundant outcomes of JAK–STAT signaling.

## Transcription factors restricted to cytokine-specific macrophage responses in vitro

In vivo, macrophages integrate signals from diverse sources, including soluble cytokines, pathogen-associated molecular patterns (PAMPs), and tissue-derived factors. To dissect the specific transcriptional responses elicited by individual signals, in vitro studies employing controlled single-stimulus experiments have proven invaluable. Historically, macrophage activation was largely conceptualized through the binary M1/M2 framework, which categorized macrophages into classically activated (M1) and alternatively activated (M2) states. However, the development of transcriptomic approaches revealed that macrophage activation states are not discrete categories but instead exist along a continuous spectrum of transcriptional states shaped by diverse environmental cues (Murray et al, 2014; Xue et al, 2014). This approach enables systematic investigation of TFs uniquely activated or required by specific stimuli, revealing the molecular logic underlying stimulus-specific macrophage activation. While these models necessarily simplify the complex, multi-signal environment macrophages encounter in vivo, they provide mechanistic insights that inform our understanding of how cells prioritize and integrate competing signals in physiological contexts. This section examines the TF networks activated by three major classes of macrophage stimuli, interferon (IFN), interleukin (IL), and Toll-like receptor (TLR)-mediated signals, which together represent some of the best-characterized signaling pathways governing macrophage activation.

Within the hierarchical framework introduced above, these in vitro systems primarily capture the activity of primary SDTFs, such as NF-κB and STATs, which act on a pre-configured enhancer landscape, and reveal an additional layer of regulation, in which secondary SDTFs further reinforce and refine transcriptional responses (Box 3). All studies discussed below, unless otherwise stated, are performed in BMDMs. Despite lacking a “tissue identity”, set in place by the collaborative action of lineage-determining and tissue-restricted TFs, that characterizes TRMs, BMDMs serve as an easily accessible, scalable, and flexible model to study the mechanistic details of signal-induced macrophage responses.

Box 3 Primary and secondary SDTFs Signal-determining transcription factors (SDTFs) are recruited to the primed enhancer landscape in a temporally structured manner. SDTFs, including NF-κB and STATs, are activated downstream of environmental cues such as microbial ligands or cytokines. Their binding drives enhancer activation through recruitment of co-activators, increased chromatin accessibility, and deposition of H3K27ac, initiating early transcriptional responses (Ghisletti et al, 2010).

A second wave of regulation is mediated by secondary SDTFs (Huisman et al, 2026), whose expression is induced by primary SDTF activity. These factors reinforce and extend transcriptional programs by targeting both pre-existing enhancers and newly formed regulatory elements. In doing so, secondary SDTFs can collaborate with LDTFs and primary SDTFs to regulate enhancer activation, particularly under conditions of sustained or complex stimulation.

Thus, macrophage gene regulation emerges from a hierarchical framework in which LDTFs define the enhancer repertoire and cellular identity, while sequential waves of primary and secondary SDTFs interpret environmental cues. This layered regulatory architecture enables both rapid and highly specific transcriptional responses, underpinning the functional plasticity of macrophages.

### Interferon-mediated transcriptional regulation

Type I interferons (IFN-I), which include IFN-α and IFN-β, signal through the heterodimeric IFNAR composed of IFNAR1 and IFNAR2, coupled to TYK2 and JAK1, respectively. Engagement of IFNAR triggers the formation of the ISGF3 transcriptional complex, consisting of STAT1, STAT2, and IRF9. The primary SDTF complex ISGF3 binds to IFN-stimulated response elements (ISREs) to drive transcription of ISGs (Platanias, 2005). Among the TFs induced by IFN-I, ATF3 has been identified as a selective repressor of a subset of ISGs. Notably, ATF3 induction is specific to IFN-I and does not occur in response to type 2 interferons (IFN-II) (Labzin et al, 2015). However, the mechanistic basis of ATF3 function remains incompletely understood, and little is known about additional IFN-I-specific secondary SDTFs, highlighting a gap in our understanding of how type I IFN responses are fine-tuned. In contrast, downstream regulatory mechanisms of IFN-II signaling have been more extensively characterized and will be discussed in greater detail below.

IFN-II, represented by IFN-γ, signals through the IFNGR receptor complex, activating JAK1 and JAK2, which phosphorylate STAT1. Phosphorylated STAT1 homodimerizes to form the gamma-activated factor (GAF), which binds to gamma-activated sequence (GAS) motifs to initiate gene transcription (Darnell et al, 1994; Ehret et al, 2001; Schindler et al, 2007). Similar to ISGF3, STAT1/GAF acts as a primary SDTF, driving the early transcriptional response to IFN-γ. Although IFN-I and IFN-II activate distinct primary SDTF complexes, both converge on overlapping sets of ISGs and induce similar chromatin remodeling at these loci, yet their downstream effects diverge due to differences in TF recruitment and regulatory dynamics (Platanitis et al, 2022; Raza et al, 2014). Notably, the early transcriptional response to IFN-I and IFN-II is highly similar; however, temporal regulation distinguishes the two programs. Genes are induced by both IFN types, but their expression is more transient following IFN-I signaling and more sustained in response to IFN-II. IRF1 provides a clear example of this differential regulation. Although IFN-I can also induce IRF1, this induction is transient, whereas IFN-II elicits a more sustained IRF1 response (Ravi Sundar Jose Geetha et al, 2024).

**Figure 2:**
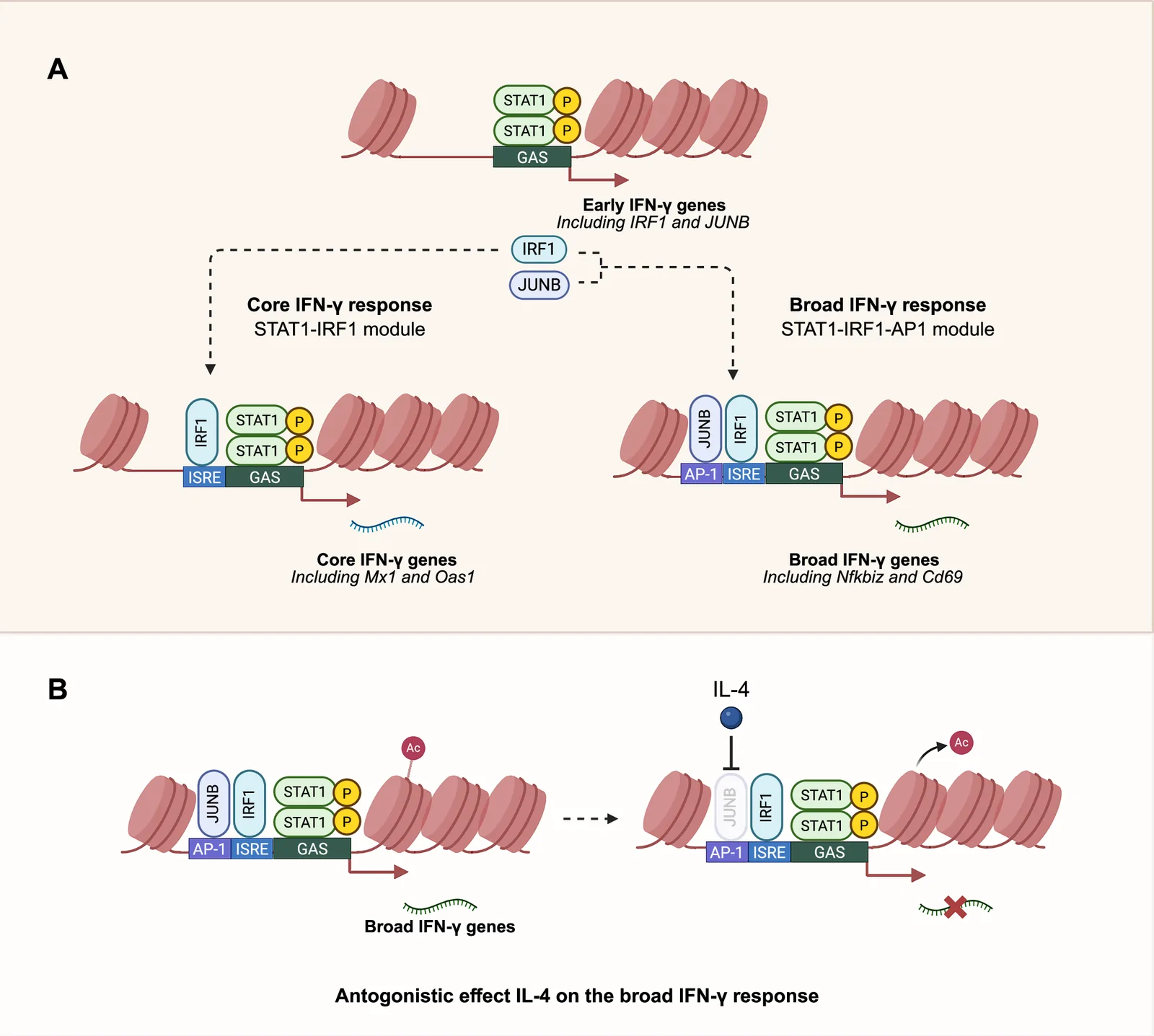
Regulation of IFN-γ-induced transcriptional programs in macrophages. (**A**) IFN-γ stimulation activates STAT1, leading to its phosphorylation and binding to gamma-activated sequence (GAS) elements and induction of early target genes, including *Irf1* and *Junb*. These factors establish two downstream modules: a core IFN-γ response driven by STAT1–IRF1 at interferon-stimulated response elements (ISRE), inducing genes such as *Mx1* and *Oas1*, and a broad IFN-γ response driven by STAT1–IRF1–AP-1 complexes at composite AP-1/ISRE sites, inducing genes such as *Nfkbiz* and *Cd69*. (**B**) IL-4 selectively antagonizes the broad IFN-γ response. While STAT1 activation and core IFN-γ gene expression remain largely preserved, IL-4 inhibits transcription at AP-1/ISRE-regulated loci, thereby suppressing induction of broad IFN-γ target genes. Illustrations were created with BioRender.

IFN-γ-mediated macrophage activation depends on secondary SDTFs acting downstream of STAT1. ATAC-seq and motif analyses identified IRF1 as a central regulator of IFN-γ-induced inflammatory macrophage polarization, working in concert with STAT1 to drive pro-inflammatory and antimicrobial programs (Chu et al, 2021) (Fig. 2A). The critical role of IRF1 in this context is underscored by human genetic and murine studies showing that IRF1 deficiency impairs macrophage responses to IFN-γ. IRF1-deficient macrophages display reduced ISG expression, diminished mycobacterial killing, and overall compromised antimicrobial activity, while their responses to IFN-α/β remain intact, indicating functional separation between IFN-I and IFN-II pathways (Rosain et al, 2023).

Further mechanistic insight was provided by Chávez et al, who demonstrated that IRF1 is essential for the formation of de novo enhancers upon IFN-γ stimulation (Chavez et al, 2025). In *Irf1*^−^^/^^−^ BMDMs, over 80% of IFN-γ-induced enhancers failed to form, revealing that IRF1 is indispensable for enhancer remodeling at most loci. While GAF alone can remodel a limited number of pre-accessible sites, broader enhancer establishment requires IRF1, which often co-localizes with STAT1 at GAS and ISRE motifs. This co-occupancy suggests sequential or cooperative binding, with IRF1 acting as a secondary SDTF after initial chromatin priming by STAT1.

Collectively, IFN signaling demonstrates how distinct primary SDTFs initiate cytokine-specific transcriptional programs that are further refined by secondary regulators. While IFN-I and IFN-II share overlapping early transcriptional responses, their downstream effects diverge through differences in the induction, timing, and function of secondary SDTFs. In particular, IRF1 is essential for sustaining IFN-γ-mediated macrophage activation and enhancer remodeling, whereas the secondary regulatory network downstream of IFN-I remains incompletely understood, highlighting an important area for future research.

### Interleukin-mediated transcriptional regulation

In contrast to IFNs, interleukins represent a functionally diverse family of cytokines that orchestrate immune cell differentiation, activation, and the resolution of inflammation. Several interleukins directly regulate macrophage polarization by engaging distinct transcriptional programs. Among these, IL-4 is the most extensively characterized driver of alternative macrophage activation, whereas IL-10 and IL-6 illustrate how different interleukin signals can drive distinct macrophage states despite both engaging STAT3-dependent pathways. Although numerous additional interleukins contribute to immune regulation, their effects on macrophage function and underlying signaling mechanisms remain less well-defined and are therefore beyond the scope of this discussion.

Engagement of IL-4Rα activates JAK1/JAK3 signaling, leading to STAT6 phosphorylation and rapid nuclear translocation. STAT6 acts as a primary SDTF, binding pre-established macrophage enhancers defined by PU.1, AP-1, and C/EBP occupancy (Czimmerer et al, 2018). Genes characteristic of the IL-4-induced macrophage phenotype include *Arg1, Mrc1, Fizz1*, and *Ym1*.

The essential role of STAT6 in this process is demonstrated by the observation that STAT6-deficient mice exhibit phenotypes closely resembling those of IL-4-deficient mice. Notably, STAT6 binding to chromatin peaks at 1 h after IL-4 stimulation, whereas STAT6-dependent transcriptional changes persist up to 24 h (Czimmerer et al, 2018). These findings suggest that additional secondary SDTFs act downstream or in concert with STAT6 to establish the full IL-4-induced macrophage phenotype. Among these, EGR2, PPARγ, and KLF4 have been identified as key contributors, whose specific roles will be discussed in detail below.

**Figure 3:**
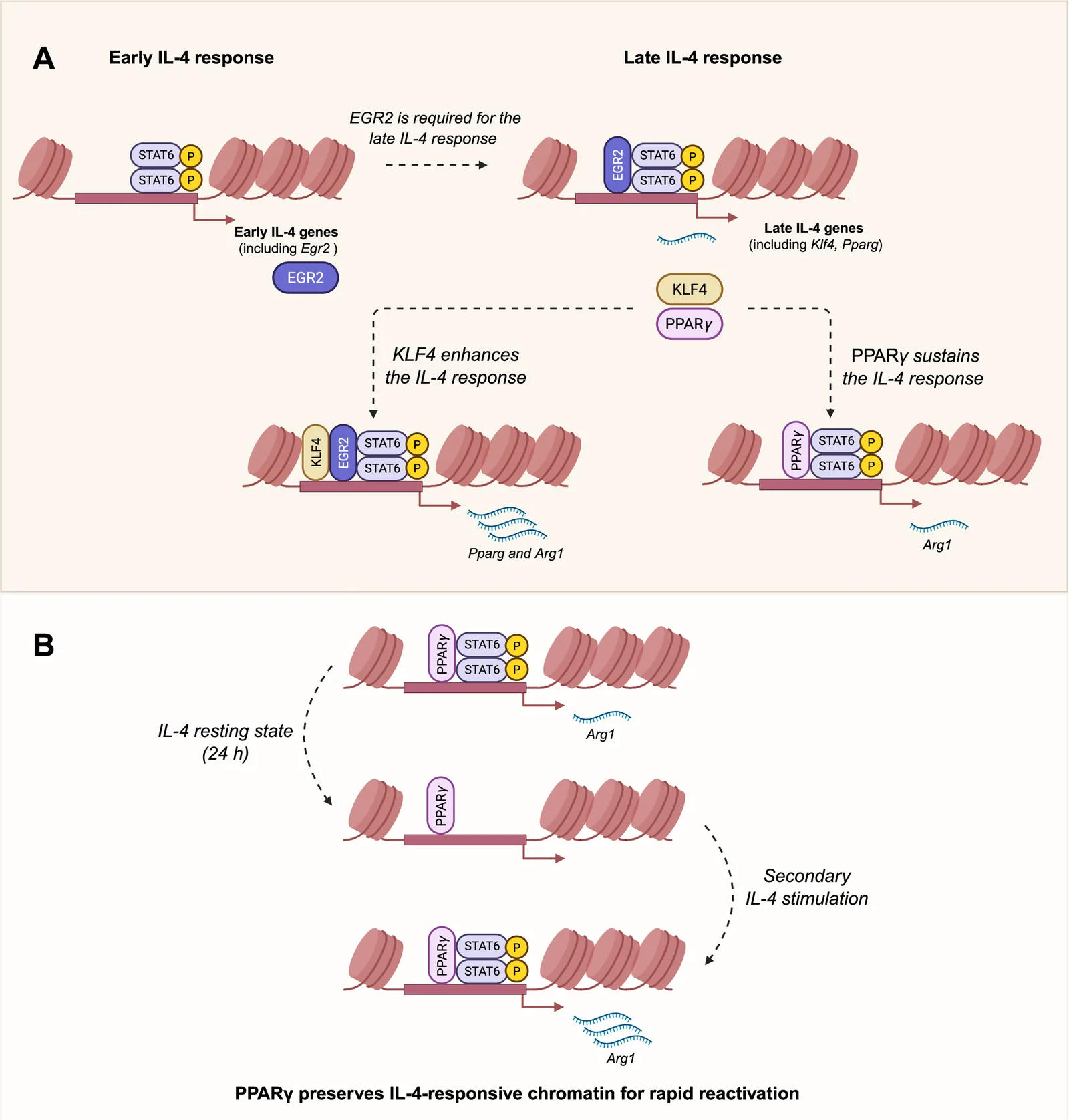
Regulation of IL-4-induced transcriptional programs in macrophages. (**A**) IL-4 stimulation initially activates STAT6, driving a primary transcriptional program that includes induction of Egr2. EGR2 is required for the transition to a secondary IL-4 response by enabling activation of additional transcription factors, including KLF4 and PPARγ. KLF4 amplifies STAT6-dependent transcription at a subset of IL-4–responsive loci, promoting expression of late IL-4 target genes such as *Pparg* and *Arg1*. PPARγ subsequently stabilizes and sustains IL-4–induced transcription, contributing to maintenance of the alternative activation program. (**B**) PPARγ mediates transcriptional memory of IL-4 stimulation. Following withdrawal of IL-4, PPARγ remains associated with IL-4–responsive regulatory regions. Upon secondary IL-4 exposure, STAT6 is rapidly reactivated, resulting in enhanced and accelerated induction of IL-4 target genes, exemplified by *Arg1*. Illustrations were created with BioRender.

The involvement of EGR2 in the IL-4-induced phenotype is supported by findings showing that EGR2 knockdown results in downregulation of hallmark IL-4-responsive genes, including *Arg1, Ym1*, and *Fizz1*. Genomic analyses reveal that early transcriptional activation regions are enriched for STAT6 motifs, whereas EGR2 motifs are enriched in regions linked to late activation (Daniel et al, 2020). Consistent with this, STAT6 binding peaks rapidly following IL-4 stimulation and subsequently declines, while EGR2 binding progressively increases, reaching its peak at 24 h. Additionally, newly acquired EGR2 binding sites correlate with elevated H3K27ac and RNA polymerase II occupancy over time, reinforcing the critical role of EGR2 in maintaining late enhancer activity (Hoeksema et al, 2021). These findings support the notion that EGR2 serves as a crucial link between the early and late phases of the IL-4 response, progressively assuming the regulatory role of STAT6 in controlling genes essential for IL-4-mediated activation (Fig. 3A). Moreover, EGR2 is required for the activation of 50 downstream IL-4-induced TFs, including PPARγ and KLF4 (Daniel et al, 2020). Time-course analyses reveal that PPARγ binding peaks at 24 h following IL-4 stimulation and remains associated with enhancers of key target genes, such as *Arg1*, even up to four days later (Fig. 3B). Collectively, these data indicate that PPARγ functions to prime chromatin for a robust secondary IL-4-induced response (Daniel et al, 2018a; Daniel et al, 2018b).

Functional studies in KLF4-deficient mice demonstrate a significant reduction in canonical IL-4 target gene expression, confirming its critical role. Luciferase reporter assays show that mutation of either KLF4 or STAT6 binding sites in the *Arg1* enhancer diminishes IL-4 responsiveness, whereas co-expression of KLF4 and STAT6 synergistically enhances transcription. KLF4 recruitment to target enhancers does depend on STAT6, while STAT6 binding occurs independently of KLF4 (Liao et al, 2011).

In contrast to the activating program induced by IL-4, IL-10 primarily functions to suppress inflammation. IL-10 signals through JAK1 and TYK2, leading to STAT3 activation, which mediates its immunosuppressive effects (Murray, 2006). Unlike IFNs, IL-10 alone induces a relatively small set of genes, reflecting its modulatory rather than activating function. Consequently, IL-10-dependent transcriptional programs are often studied in the context of an inflammatory co-stimulus, making it challenging to isolate IL-10-specific TFs. Notably, STAT3 does not directly repress inflammatory genes, implying that IL-10 acts through indirect or epigenetic mechanisms. Supporting this, recent evidence in human MDMs suggests that IL-10 suppresses IFN responses by preventing LPS-induced chromatin remodeling, potentially by inhibiting the expression and activity of IRFs. Upon co-stimulation with LPS, IL-10 reduces chromatin accessibility at ISG loci, leading to decreased ISG expression. Specifically, IRF motif occupancy in the accessible sites is completely diminished with IL-10 and LPS stimulation compared to LPS alone, and 90% of LPS-induced IRF1 binding peaks are lost during IL-10 co-stimulation, although IL-10 does not affect basal IRF1 binding. Mechanistically, IL-10 reduces chromatin accessibility by modulating histone acetylation associated with IRF1, but it does not induce epigenomic changes on its own to explain the repressed IFN signaling observed during subsequent LPS stimulation (Mishra et al, 2025).

Besides IL-10, IL-6 is a well-known activator of STAT3, yet the cellular outcomes of IL-6 and IL-10 signaling differ markedly. SOCS3 appears to be a key discriminator between the two pathways, as it is induced in similar patterns by both cytokines but preferentially inhibits IL-6 signaling (Yasukawa et al, 2003). IL-6 triggers a rapid but relatively short-lived wave of STAT3 phosphorylation that is efficiently terminated as SOCS3 accumulates and binds gp130, sharply limiting the duration of IL-6-STAT3 signaling. In contrast, IL-10-induced STAT3 activation in macrophages is more prolonged and remains active despite high SOCS3 levels, because SOCS3 does not efficiently bind or shut down the IL-10 receptor complex (Croker et al, 2012). How these distinct STAT3 activation dynamics translate into differential transcriptional and functional responses remains incompletely understood.

Interleukin signaling demonstrates that macrophage phenotypes are shaped by hierarchical TF networks downstream of shared pathways. IL-4 provides a clear example in which STAT6-driven responses are reinforced by secondary SDTFs, including EGR2, PPARγ, and KLF4, to establish a stable alternative activation program. In contrast, although IL-10 and IL-6 both signal through STAT3, the secondary SDTFs that define their distinct transcriptional and functional outcomes remain largely unknown, highlighting a key area for future investigation.

### Toll-like receptor-mediated transcriptional regulation

TLRs and cytokine receptors activate overlapping but not identical signaling programs, and the nature of the upstream stimulus strongly influences the downstream epigenomic response. In macrophages, different TLRs engage distinct adapter combinations: TLR4 is unique in signaling through both MyD88 and TRIF, TLR3 signals exclusively through TRIF, and most other TLRs primarily use MyD88. These differences shape the balance between NF-κB, AP-1, and IRF-dependent outputs, and therefore determine not only the immediate transcriptional response but also the extent of chromatin remodeling.

NF-κB activity is tightly controlled by inhibitory IκB proteins and feedback loops that regulate nuclear translocation and DNA binding (Dorrington and Fraser, 2019). Although many receptors converge on the canonical NF-κB pathway, the upstream signaling context determines how long NF-κB remains nuclear, which dimers are engaged, and how effectively it cooperates with other TFs. As a result, NF-κB activation is not a uniform output but a highly tunable regulatory module that integrates receptor identity, signaling strength, and feedback control.

Differences in NF-κB dynamics are particularly evident when comparing LPS, poly(I:C), and TNF (Vallabhapurapu and Karin, 2009). All three activate NF-κB, but through distinct signaling routes. LPS, via TLR4, engages both MyD88 and TRIF pathways, resulting in a strong and sustained NF-κB response. In contrast, poly(I:C), acting through TLR3, signals only through TRIF, while TNF activates NF-κB through a cytokine receptor pathway that lacks TLR-specific adapter structure. These differences do not prevent inflammatory gene induction, but they do affect how NF-κB behaves over time.

This temporal behavior is critical for epigenomic regulation. NF-κB-driven chromatin remodeling depends not simply on whether NF-κB is activated, but on how it is activated. Non-oscillatory NF-κB is substantially more effective than oscillatory NF-κB at promoting chromatin accessibility and H3K4me1 deposition at latent enhancers (Cheng et al, 2021). In their stimulus comparisons, TNF and poly(I:C) produced much less enhancer formation than LPS and other pathogen-derived ligands, consistent with the idea that sustained NF-κB dynamics are required for robust epigenomic reprogramming. More broadly, this supports the idea that stimulus identity is encoded in the temporal structure of NF-κB activation rather than in pathway engagement alone.

In parallel, activation of the MyD88-dependent pathway also induces AP-1 family TFs through MAPK signaling pathways. AP-1 activity emerges from the combinatorial assembly of JUN, FOS, and ATF family members into various dimers whose composition is determined by stimulus-specific regulation of their expression and phosphorylation (Shaulian and Karin, 2002). Unlike NF-κB, whose activity is strongly shaped by oscillatory dynamics, AP-1 activation is more closely tied to kinase-driven phosphorylation events that regulate both subunit abundance and transactivation potential (Kawai and Akira, 2006). AP-1 plays a key role in shaping chromatin accessibility, particularly at enhancers, where it often cooperates with LDTFs such as PU.1 to establish or stabilize accessible regulatory regions. Importantly, AP-1 frequently acts in concert with NF-κB at composite regulatory elements, and their cooperative binding can enhance both transcriptional output and chromatin remodeling (Zhao et al, 2011). However, the extent, timing, and subunit composition of AP-1 activation vary across stimuli, contributing an additional layer of specificity to the epigenomic response

In the case of TLR3 and TLR4 activation, the NF-κB response is coupled to TRIF-dependent activation of IRF3, allowing the pathway to move from immediate inflammatory signaling into an IFN-producing program that further amplifies and diversifies the response. The TRIF-dependent branch directly activates IRF3. Upon LPS stimulation, IRF3 is rapidly phosphorylated, dimerizes, and translocates to the nucleus, where it binds regulatory elements at the *Ifnb1* locus to initiate transcription of IFNβ. This early IFN-β production establishes an autocrine/paracrine signaling loop that activates ISGF3, thereby amplifying the IFN response and inducing ISGs.

Efficient induction of *Ifnb1* also requires IRF8, which is constitutively bound at the promoter together with PU.1 (Mancino et al, 2015). IRF8 facilitates recruitment of IRF3 and is essential for full transcriptional activation. Macrophages lacking functional IRF8 show impaired *Ifnb1* induction and reduced downstream STAT1 phosphorylation and IRF1 expression, despite intact NF-κB activation and IRF3 phosphorylation. This indicates that IRF8 is dispensable for upstream signaling but required to establish transcriptional competence at the locus.

While IRF3 mediates the immediate response to LPS, IRF1 operates at a later stage (Chavez et al, 2025). In contrast to IRF3, which is directly activated downstream of TLR4 signaling, IRF1 is transcriptionally induced downstream of NF-κB and IFN signaling. As discussed in detail above, IRF1 drives the formation of de novo enhancers at ISGs. Through this function, IRF1 amplifies and sustains the transcriptional program initiated by IRF3, linking early pathogen sensing to long-term transcriptional reprogramming.

Together, these findings support a hierarchical model of TLR4 signaling in which the primary SDTFs NF-κB and IRF3 initiate rapid gene induction, while secondary SDTFs add an additional regulatory layer: IRF8 establishes locus competence, and IRF1 drives widespread enhancer formation to sustain and expand the transcriptional response. More generally, they suggest that the combination of receptor usage, signaling dynamics, and TF cooperation determines whether a stimulus produces a transient inflammatory burst or durable epigenomic rewriting.

### Opposing and synergistic transcriptional crosstalk

In vivo, macrophages are exposed to a diverse and dynamic array of environmental cues. These signals, often concurrent and even opposing, shape macrophage identity in complex ways that cannot be fully captured by single-stimulus models. Consequently, in vitro studies exploring the combinatorial effects of multiple signals have provided critical insights into how macrophages integrate and prioritize competing transcriptional programs.

A well-characterized example of antagonistic signaling is provided by IFN-γ and IL-4, which drive opposing polarization states. Piccolo et al showed that subsets of genes induced by one cytokine are selectively repressed by the other in a dose-dependent manner, with prior IL-4 exposure dampening subsequent IFN-γ responses, indicating short-term transcriptional memory (Piccolo et al, 2017). Mechanistically, at the level of *cis*-regulatory architecture, the sensitivity of IFN-γ-induced genes to IL-4 depended on enhancer complexity. Genes controlled by simple elements containing only core IFN-γ-responsive motifs (GAS and IRF sites) were largely resistant to IL-4-mediated repression. In contrast, IL-4 selectively inhibited genes controlled by more complex enhancers that required additional secondary SDTFs, like JUNB, an AP-1 family TF. (Kang et al, 2017) JUNB is induced downstream of IFN-γ and contributes to enhancer activation by promoting deposition of the active histone mark H3K27ac at IFN-γ-responsive regulatory regions. IL-4 co-stimulation reduces JUNB binding and leads to diminished H3K27ac at these enhancers, selectively impairing this subset of IFN-γ-responsive genes, while leaving core antimicrobial programs intact. Conversely, IFN-γ broadly suppresses IL-4-induced chromatin remodeling and STAT6 binding (Fig. 2B).

However, macrophage responses to combined signals are not purely antagonistic and display substantial heterogeneity. Under simultaneous stimulation with IFN-γ, IL-4, and LPS, population-level analyses suggest mutual repression of canonical polarization markers (Muñoz-Rojas et al, 2021). Yet, single-cell RNA-seq reveals diverse responses, with subsets of macrophages maintaining IL-4 responsive gene expression (e.g., *Arg1, Socs2, Mafb*, and *Klf4*), highlighting variability in sensitivity to competing cues.

**Figure 4:**
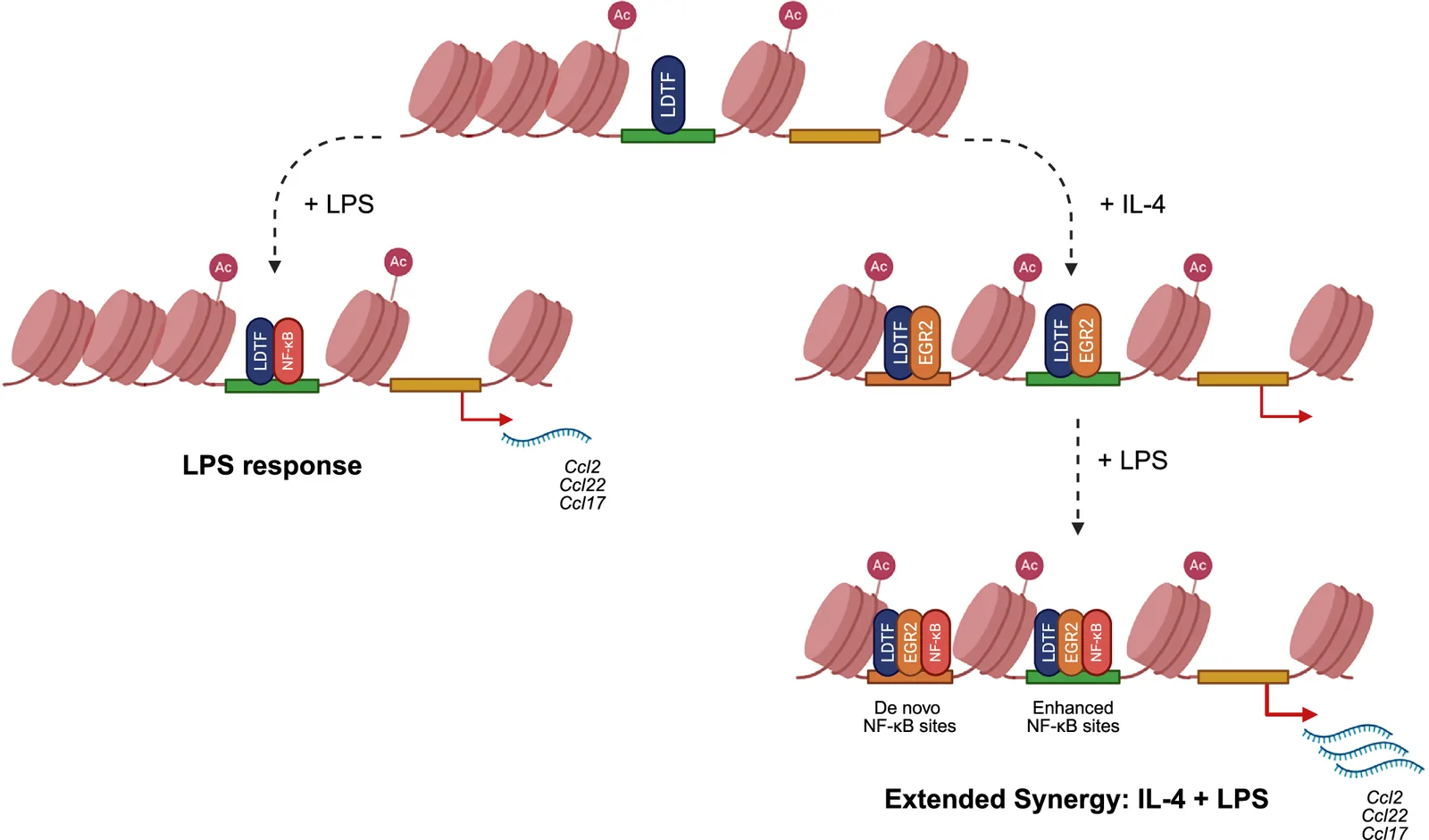
IL-4 priming enables extended synergy with LPS in macrophages. In naïve macrophages, LPS stimulation activates NF-κB, driving transcription at a limited set of accessible enhancers and inducing moderate expression of target genes such as *Ccl2*, *Ccl22*, and *Ccl17*. IL-4 stimulation activates STAT6, leading to EGR2-dependent chromatin remodeling and enhancer activation. Upon subsequent LPS exposure, NF-κB is recruited to both pre-existing and de novo enhancers, resulting in enhanced and expanded transcriptional responses and amplified expression of a subset of inflammatory genes. Illustrations were created with BioRender.

Importantly, the temporal sequence of signals can fundamentally alter outcomes. Sequential exposure to IL-4 followed by LPS induces strong and sustained synergy rather than antagonism (Czimmerer et al, 2022) (Fig. 4). IL-4 priming drives STAT6-dependent epigenomic reprogramming, increasing chromatin accessibility, enhancer activation, and cofactor recruitment. This leads to a marked expansion of the NF-κB cistrome, with thousands of newly accessible binding sites, and results in a dramatic amplification of LPS-induced transcription for a defined subset of genes. These include both genes already responsive to LPS and genes that are induced de novo only in the primed state, illustrating how prior exposure can qualitatively reshape inflammatory responses. Mechanistically, EGR2 is essential for IL-4-mediated chromatin remodeling and for achieving full synergistic activation at a subset of enhancers.

A similar priming effect is observed with IFN-γ. Priming with IFN-γ markedly enhances LPS-induced expression of pro-inflammatory cytokine genes, including *Tnf* and *Il12b*, by increasing H3K27ac at distal regulatory elements in both human monocytes and murine macrophages (Qiao et al, 2013). This priming effect reflects IFN-γ-dependent chromatin remodeling that establishes a more permissive enhancer landscape for TLR signaling. Notably, this response augments a subset of inflammatory genes, while other genes, such as *Il10*, are repressed. IFN-γ priming promotes recruitment of LDTFs and SDTFs to latent enhancers, effectively pre-loading regulatory regions for rapid activation upon LPS stimulation. In human macrophages, IFN-γ-mediated priming of the *Tnf* locus is driven by IRF1, as IFN-γ-induced IRF1 binding is required for the enhanced response to LPS (Chow et al, 2014).

Different TLRs can also alter subsequent TLR responses (Wang et al, 2025). Prior exposure to ligands like poly(I:C) (via TLR3/TRIF) or CpG (via TLR9/MyD88) distinctly alters subsequent NF-κB responses to TNF or LPS. Notably, poly(I:C) pretreatment produced a priming effect, increasing activation amplitude and duration, while CpG induced tolerance, attenuating later responses. These memory effects were reflected in altered NF-κB amplitude, duration, and single-cell response trajectories. This inflammatory memory is not only mediated by altered NF-κB dynamics but also by changes in chromatin accessibility. Chromatin accessibility is rapidly remodeled following inflammatory stimulation, with stimulus-specific opening of promoters and enhancers shaping transcriptional responses. Poly(I:C) and CpG induce distinct, yet partially overlapping, accessibility landscapes enriched for key inflammatory TF motifs, including NF-κB (RelA) and AP-1, with additional enrichment of ISREs in poly(I:C)-treated cells and C/EBP motifs in CpG-treated cells. Moreover, chromatin accessibility patterns established by the initial stimulus are largely resistant to subsequent signals. Even after secondary stimulation with TNFα or LPS, the accessibility landscape more closely resembles that induced by the first stimulus, indicating that early inflammatory cues dominantly imprint the epigenetic state.

While IFN-γ/LPS and IL-4 exemplify opposing cytokine signals, not all cytokine interactions are antagonistic. In human MDMs, co-stimulation with IL-4 and IL-6 generates both synergistic and antagonistic transcriptional responses (Jura et al, 2008). IL-6 alone failed to repress IL-4-induced genes, whereas IL-4 antagonized approximately one-quarter of IL-6 target genes. Interestingly, co-stimulation led to elevated expression of the TF BATF compared with either cytokine alone. Silencing BATF selectively impaired expression of co-induced genes, identifying BATF as a potential integrator of IL-4/IL-6 signaling. Similar synergy is observed with IL-4 and IL-10, where co-stimulation amplifies canonical anti-inflammatory macrophage gene expression, including *Arg1*, and IL-10 reinforces IL-4 responsiveness by upregulating IL-4Rα (Makita et al, 2015). Given that IL-6 and IL-10 both activate STAT3, these effects likely reflect cooperative STAT3-STAT6 signaling. Indeed, simultaneous activation of STAT3 and STAT6 synergistically enhances target gene expression, a response lost upon deletion of either factor (Yan et al, 2016).

A comparable principle applies to co-stimulation with IL-6 and IFN-γ. Rather than uniformly modulating IFN-γ responses, IL-6 selectively reshapes gene expression, with subsets of IFN-γ–induced genes being further enhanced, attenuated, or unaffected. This gene-specific regulation is accompanied by concurrent activation of STAT1 and STAT3 and increased STAT1–STAT3 genomic co-occupancy. Genes that are upregulated, particularly those most strongly enhanced by co-stimulation, are enriched for STAT1–STAT3 co-binding, whereas genes with reduced induction show little overlap. Together, these observations highlight STAT convergence as a central mechanism by which cytokine combinations fine-tune macrophage transcriptional programs toward context-dependent inflammatory or tissue-repair states (Botman et al, 2026).

Another important signal impacting macrophage phenotype is glucocorticoid (GC) signaling. Similar to IL-4 stimulation, GC-treated macrophages acquire anti-inflammatory characteristics associated with phagocytosis, wound healing, and tissue repair, suggesting convergence between these distinct pathways (Deochand et al, 2024). GCs activate the glucocorticoid receptor (GR), which shows substantial genomic overlap with STAT6 binding sites in macrophages, indicating cooperative regulation of shared enhancers (Caratti et al, 2023; Deochand et al, 2024). Combined IL-4 and GC stimulation produces additive and synergistic induction of anti-inflammatory genes, including *Mrc1, Arg1*, and *Klf4*, accompanied by increased H3K27ac deposition at regulatory regions. Loss of GR reduces STAT6 recruitment to these enhancers, demonstrating that GR facilitates STAT6-dependent enhancer activation (Caratti et al, 2023). Thus, GC signaling reinforces IL-4-driven macrophage polarization through chromatin-level cooperation between GR and STAT6. In addition to reinforcing IL-4-driven anti-inflammatory programs, GC signaling also modulates inflammatory responses triggered by TLR activation. During TLR stimulation, GR is recruited to a subset of NF-κB-bound regulatory elements in a ligand-dependent manner without displacing NF-κB from chromatin (Chinenov et al, 2012). Instead, GR interacts with NF-κB-associated regulatory complexes to suppress transcriptional activation at inflammatory loci. Thus, GR integrates distinct macrophage signaling environments by promoting anti-inflammatory gene programs in cooperation with STAT6 while restraining NF-κB-dependent inflammatory transcription.

Taken together, these studies support the view that macrophage activation in vivo is not a simple on/off process but rather a continuum of transcriptional states shaped by the balance, timing, and sequence of cytokine and microbial inputs. Within the hierarchical TF framework, these signals are integrated through context-dependent modulation of SDTF activity, enhancer accessibility, and TF cooperation. Antagonistic interactions, such as IFN-γ and IL-4, selectively disrupt competing SDTF-dependent regulatory programs, whereas synergistic interactions, including IL-4 with LPS, IL-6 or IL-10, promote cooperative SDTF recruitment and chromatin remodeling. Similarly, sequential exposure to inflammatory stimuli such as poly(I:C)/CpG or IFN-γ/LPS establishes distinct forms of transcriptional memory by reshaping enhancer landscapes and subsequent TF responses. Thus, macrophage phenotypic diversity is determined not only by the identity of the signals encountered but also by their temporal integration through hierarchical TFs networks.

## Disease implications and therapeutic potential

Maintaining tissue homeostasis requires tightly controlled immune responses that are coordinated by cytokines, chemokines, and other signaling mediators released within the microenvironment. Under physiological conditions, these signals instruct appropriate transcriptional programs that support host defense and resolution of inflammation. Persistent dysregulation of cytokine signaling in the diseased tissue not only prolongs inflammation, but it also actively reprograms myeloid differentiation and macrophage identity at tissue sites. Chronic exposure to cytokines like TNF, IL-6 family cytokines, IL-1β, IL-23, GM-CSF, and type I IFNs leads to sustained activation of SDTFs in monocytes and tissue-resident macrophages. Within the hierarchical regulatory framework described above, this persistent signaling drives stable reprogramming of macrophage enhancer landscapes and transcriptional networks, causing macrophages to diverge from homeostatic functions. As a result, macrophages adopt pathogenic transcriptional states that reinforce disease progression, commonly referred to as disease-associated macrophages (DAMs).

Although the distinction between primary and secondary SDTFs provides a useful framework for dissecting transcriptional mechanisms, it becomes less clear during chronic inflammation. Persistent cytokine and PRR signaling activates multiple SDTFs, creating feed-forward transcriptional circuits that stabilize disease-associated enhancer landscapes. As a result, cytokine-responsive TFs act together as disease-associated SDTFs, cooperating with tissue-specific lineage-defining and tissue-resident TFs to establish and maintain pathogenic macrophage identities.

### Rheumatoid arthritis

Rheumatoid arthritis (RA) offers a useful example of how such pathogenic transcriptional states emerge in a disease-specific manner. In RA, circulating monocytes and incoming precursors encounter a synovial microenvironment enriched in TNF, IL-6, GM-CSF, IFNs, and immune complexes, which together drive their differentiation into macrophages with aberrant inflammatory, metabolic, and tissue-remodeling programs. Single-cell and spatial transcriptomic studies have revealed substantial heterogeneity among synovial macrophages, identifying distinct subsets associated with either active inflammation or disease remission. A macrophage population enriched at the pannus-cartilage interface expresses inflammatory and tissue-destructive gene programs, whereas a distinct lining-layer subset observed in patients in remission expresses TFs such as KLF4 and NR4A2. Expression of these factors negatively correlates with disease activity and is thought to support regulatory or resolving macrophage phenotypes (Alivernini et al, 2020; Mitsui and Satoh, 2025).

Multiple TFs have been described to be involved in RA, including STAT3, STAT1, and IRF1, and thus could be potential disease-associated SDTFs. Sustained IL-6 signaling promotes STAT3 activation, driving metabolic remodeling, enhanced inflammatory mediator production, and stabilization of hyperinflammatory macrophage programs. STAT3 inhibition in MDMs reduces their hyperinflammatory phenotype, although the relative contribution of STAT3-dependent programs in human RA tissue requires further validation (Hanlon et al, 2023).

In addition to STAT3, IFN-driven activation of STAT1 and IRF1 contributes to inflammatory macrophage states, suggesting that multiple disease-associated SDTF networks cooperate rather than acting through a single dominant regulatory pathway. Consistent with this, single-cell transcriptomic analyses of the human RA synovium identified distinct inflammatory myeloid states, including an infiltrating monocyte-derived STAT1⁺CXCL10⁺ population characterized by IFN-response gene expression and an IL1B⁺FCN1⁺HBEGF⁺ inflammatory monocyte state (Zhang et al, 2023). A cross-disease integration of single-cell datasets demonstrated that related inflammatory macrophage programs are conserved across RA, inflammatory bowel disease (IBD), and severe COVID-19, identifying CXCL10⁺CCL2⁺ and FCN1⁺ inflammatory macrophage populations across tissues (Zhang et al, 2021). The CXCL10⁺CCL2⁺ state showed enrichment of IFN-responsive genes, including *STAT1*, *IRF1*, and *CXCL11*, whereas FCN1⁺ macrophages expressed inflammatory alarmins such as *S100A8* and *S100A9*. Ex vivo stimulation experiments further showed that IFN-γ together with TNF can induce the CXCL10⁺CCL2⁺ macrophage program, linking inflammatory cytokine environments to activation of conserved STAT/IRF and NF-κB-associated SDTF networks. Supporting these findings, single-cell chromatin accessibility profiling of RA myeloid cells identified five regulatory states, including two tissue-resident macrophage populations (LYVE1⁺TIMD4⁺ and F13A1⁺MARCKS⁺ TRM) and an infiltrating inflammatory monocyte population (FCN1⁺SAMSN1⁺) overlapping with the previously described pro-inflammatory states (Weinand et al, 2024). These chromatin states were associated with distinct TF motif enrichments: KLF motifs, including KLF4, were enriched in LYVE1⁺TIMD4⁺ resident macrophages, consistent with the increased KLF4 expression observed in remission-associated macrophage populations, whereas AP-1 and CEBP motifs were enriched in inflammatory monocyte and intermediate macrophage states.

Given the central role of JAK–STAT signaling in shaping these disease-associated SDTF networks, it is not surprising that JAK inhibitors are effective therapies in RA. Treatment with tofacitinib reduces STAT1 expression in macrophage populations associated with high disease activity (Xia et al, 2025). Other TFs associated with higher disease activity were IRF1 and FOS. In addition, the JAK inhibitor Upadacitinib promotes a shift toward more anti-inflammatory macrophage phenotypes characterized by increased expression of MafB and MafB-associated transcriptional networks (López-Navarro et al, 2024). MafB is a key TF governing the anti-inflammatory transcriptional programs of macrophages (Kim, 2017). Similar transcriptional reprogramming is observed with other JAK inhibitors, suggesting a shared mechanism of action. Together, these findings indicate that JAK inhibition consistently reshapes disease-associated TF networks, raising the possibility that selectively targeting pathogenic TFs downstream of JAK signaling could achieve therapeutic benefit while avoiding the broad side effects of JAK inhibitors (Box 4).

Box 4 The future of cell-type-specific targeting of cytokine pathways Historically, the blockade of different constituents of the JAK–STAT pathway has been a key therapeutic strategy against a range of inflammatory disorders, due to the prevalence of aberrant cytokine signaling in their pathogenesis. Lack of technical expertise on TF targeting made Janus kinases the most attractive targets for inhibitor development. However, even highly specific targeting of one JAK will likely affect the signaling of multiple cytokine pathways and, subsequently, their biological functions. This has naturally resulted in considerable side effects associated with their use (Hoisnard et al, 2022). In addition, these inhibitors act systemically, potentially disrupting homeostatic and non-pathological functions of the signaling pathways they inhibit. As mentioned in this review, a pre-configured enhancer landscape is set in place by LDTFs, which differ across cell types. This means that targeting SDTFs will result in divergent transcriptional outputs in different cell types. Consequently, JAK inhibition is likely to alter diverse gene programs across multiple cell subsets, with outcomes that remain difficult to predict due to the incomplete characterization of these context-specific effects. In the example of RA presented in the main text, clinically approved JAK inhibitors, including tofacitinib and baricitinib, that aim to target pro-inflammatory programs, possibly mediated downstream of both IL-6 and IFN-γ that engage JAK1, will also interfere with the signaling pathways of IL-4 and IL-10 that instead promote reparative macrophage polarization. This illustrates how the lack of selectivity translates into broad and potentially counterproductive transcriptional consequences within a single-cell-type.

The development of more targeted therapeutic approaches is therefore warranted, with a focus on the following objectives:

- Investment in single-cell technologies that will characterize the aberrant gene programs and cell subsets that contribute to disease pathogenesis and progression
- Identification of secondary SDTFs that sustain these inflammatory programs in the cells of interest
- Mechanistic characterization of these transcriptional regulators
- Innovation in TF-targeting technologies
- Development of strategies for tissue- and cell-specific delivery of therapeutic cargo

### Other inflammatory diseases

An imbalance in cytokine signaling cascades within the gastrointestinal tract that favors a pro-inflammatory environment can drive the development of IBD, which primarily comprises ulcerative colitis and Crohn’s disease. Similar to RA, inflammatory cytokine and PRR signaling in IBD is expected to generate disease-associated SDTF networks that stabilize pathogenic macrophage states. Single-cell and spatial profiling studies have revealed that intestinal macrophages in IBD are highly heterogeneous, with inflammatory states markedly expanded compared with those observed in healthy tissue (Garrido-Trigo et al, 2023). In inflamed mucosa, infiltrating monocytes differentiate into two transcriptionally distinct inflammatory macrophage populations, as well as an alternative inflammation-dependent alternatively activated (IDA) macrophage state that displays features of both inflammatory and anti-inflammatory programs. These DAMs form a lineage branch distinct from resident, homeostatic macrophages. IDA macrophages are transcriptionally intermediate and highly heterogeneous, with signatures that vary substantially across patients and datasets, reflecting differences in local inflammatory cues. As a result, macrophages are exposed to distinct environmental cues and microenvironments that shape their differentiation states. Spatial mapping reveals that inflammatory macrophages cluster near sites of tissue damage, whereas IDA macrophages are more broadly distributed and frequently associate with fibroblasts, forming cellular circuits that sustain chronic inflammation. Distinct transcriptional profiles of IDA macrophages correlate with their localization within diseased tissue, highlighting the role of niche-specific signals in shaping macrophage states (Garrido-Trigo et al, 2023). The cross-disease single-cell analyses discussed above further suggest that inflammatory macrophage programs are not restricted to individual diseases but reflect conserved responses to chronic inflammatory environments (Zhang et al, 2021). However, compared with RA, where specific SDTF modules such as STAT3, STAT1, and IRF1 have been more extensively characterized, the regulatory networks maintaining IBD-associated macrophage states remain less well-defined. Persistent exposure to cytokines and microbial-derived signals, including TNF, IL-1β, IL-6, IL-23, and PRR ligands, is expected to activate interconnected STAT-, IRF-, NF-κB-, and AP-1-dependent transcriptional programs that stabilize pathogenic macrophage states. The marked heterogeneity of IBD-associated macrophages likely reflects differences in the combination and intensity of these SDTF networks together with tissue-specific regulatory inputs from the local microenvironment.

Systemic lupus erythematosus (SLE) provides another example where aberrant cytokine signaling leads to the emergence of DAMs. Patients exhibit systemic cytokine dysregulation, including elevated IFN-I, TNF, IL-1β, IL-17A, and IL-10 with IFN signatures correlating with disease severity (Duarte-Delgado et al, 2024; Mai et al, 2021). In lupus nephritis, a common complication in SLE patients, infiltrating monocytes give rise to transcriptionally distinct DAM populations that are conserved across human disease and lupus mouse models and are characterized by the expression of injury- and lipid-associated gene programs (Hoover et al, 2025). These populations are distinct from resident kidney macrophages, although resident macrophages partially acquire disease-associated features during inflammation, resulting in overlapping phenotypic states. As in IBD, DAMs in lupus nephritis display marked transcriptional heterogeneity shaped by local renal microenvironments (Hoover et al, 2025). Although the specific disease-associated SDTFs maintaining lupus-associated macrophage states remain largely undefined, the prominent IFN-I signature in SLE suggests that IFN-responsive STAT and IRF networks are likely important regulators of these pathogenic programs.

The aforementioned examples illustrate how disease-specific cytokine environments shape pathogenic macrophage states, suggesting that targeted modulation of TFs could circumvent the side effects of broad JAK–STAT inhibition.

### Therapeutic potential of targeting disease-associated macrophages

Selective targeting of transcriptional regulators driving pathogenic macrophage states presents an exciting therapeutic prospect to fine-tune inflammatory responses at a level downstream of broadly shared signaling pathways. Rather than blocking upstream cytokine signaling, which often affects multiple cell types and pathways, modulation of SDTFs and their downstream networks may enable more precise control over disease-associated gene programs. Targeting TFs that shape or maintain enhancer landscapes could allow selective rewiring of macrophage transcriptional states, shifting cells away from pathogenic programs while preserving essential immune functions (Box 4). A recent study highlighted the therapeutic potential of targeting macrophage TFs by identifying the shared molecular mechanism underlying an intergenic haplotype associated with five inflammatory diseases. The authors showed that the putative causal variant resides in a macrophage-specific enhancer regulating *ETS2* expression, with the risk allele increasing *ETS2* levels. ETS2 was found to orchestrate macrophage inflammatory programs and regulate IBD-associated genes, including *TNF* and *IL23*. As no direct ETS2 inhibitors are currently available, the authors inhibited MEK, an upstream regulator of ETS2, in intestinal biopsies from untreated IBD. MEK inhibition reduced inflammatory cytokine release to levels comparable to those achieved with clinically approved anti-TNF therapy (Stankey et al, 2024).

While the study provides compelling evidence for the therapeutic potential of targeting TF-driven inflammation, it also reveals the challenges associated with translating this approach into clinical application. TFs lack well-defined binding pockets, complicating the development of small-molecule inhibitors. Their function relies on transient protein-protein and DNA-protein interactions across large surfaces, making disruption inherently difficult (Chen and Koehler, 2020). An additional layer of complexity arises from the need to deliver any therapeutic specifically to the relevant macrophage subset. Identifying high-confidence markers unique to DAMs would require extensive screening of antigens and receptors using advanced omics and imaging technologies. Several cell-specific delivery strategies are under exploration, including nucleic acid-based approaches, monoclonal antibody–drug conjugates, and peptide-mediated systems. Each requires three components: a ligand that selectively engages the target cells, a therapeutic cargo, and a linker connecting the two (Zhao et al, 2020). Ligands can be single-stranded oligonucleotides, antibodies, or peptides recognizing surface receptors, while cargos include small molecules or antisense oligonucleotides (ASOs). ASOs are particularly promising for targeting “undruggable” TFs, as they act at the transcript level and bypass challenges associated with disrupting protein interactions (Crooke et al, 2021). Although the development of TF inhibitors and specific delivery of those to DAMs remains mostly at the stages of conceptualization and basic research, rapid advances in drug development and delivery technologies offer promising prospects for clinical success.

The study of transcriptional control of macrophages has not only demonstrated the possibility of targeting pathogenic states through the modulation of TF activity. Instead, it has also illustrated that stratification of patients based on transcriptional and epigenetic signatures is feasible with technologies already available to us and can offer a powerful tool for monitoring therapy response.

## Conclusion

The evidence reviewed here establishes that the specificity of signal responses in macrophages cannot be encoded by one or two TFs alone but is rather achieved by a network of TFs acting in a temporally- and spatially appropriate manner. Upon tissue engraftment, environmental cues shape the phenotype of developing macrophages by directing the expression of tissue-specific transcriptional regulators that act in concert with LDTFs. These establish specialized gene programs that allow TRMs to take on niche-restricted functions, including surfactant metabolism by alveolar macrophages. Signal responses in mature macrophages are mediated by signal-restricted TFs, but their specification is convoluted due to the complexity of the tissue niche that macrophages are embedded in, which provides not only diverse cues from neighboring cells, but also direct cell-cell interactions and mechanical forces of the ECM. Faced with a dynamic environment of varying external cues, macrophages continuously integrate this input through cooperative and antagonistic interactions between SDTFs, producing context-dependent transcriptional outputs.

Coupling signaling modalities to highly specialized TFs has been enabled by mechanistic studies in controlled laboratory settings that remove the confounding factors of a tissular niche. This has demonstrated, for example, that early cytokine responses controlled by STATs are progressively refined and sustained by secondary SDTFs, including EGR2, PPARγ, KLF4, and IRF1. The studies discussed also highlight that macrophages integrate concurrent and opposing cytokine signals, resulting in continuous and heterogeneous transcriptional states rather than binary polarization outcomes.

This sensitivity to environmental signals represents a double-edged sword. While it enables macrophages to adapt to tissue-specific demands, disruption of signaling cascades in disease drives ectopic TF induction and the emergence of pathogenic macrophage states. Collectively, the findings discussed here position transcriptional regulation as a central determinant of macrophage-mediated pathology and suggest that dissecting signal-restricted transcriptional mechanisms may enable more selective therapeutic strategies that move beyond broad inhibition of signaling cascades.

## Supplementary information

Peer Review File
