# Supplementary material for: Multi-level transcriptional control of specific macrophage responses
Source: EMBO J. 2026 Aug 13;45(20):7021–40. doi: 10.1038/s44318-026-00898-7 (PMC13672315; doi:10.1038/s44318-026-00898-7)
Supplement: Supplementary file 1 [file 44318_2026_898_MOESM1_ESM.pdf]

THE EMBO JOURNAL

Multi-level transcriptional control of specific macrophage responses

Alexandra Drakaki, Josje Huisman, and Marten Hoeksema

Corresponding author(s): Marten Hoeksema (m.a.hoeksema@amsterdamumc.nl)

Review Timeline:

Submission Date:	22nd May 26
Editorial Decision:	24th Jun 26
Revision Received:	22nd Jul 26
Accepted:	23rd Jul 26

Editor: Ioannis Papaioannou

Transaction Report:

(Note: With the exception of the correction of typographical or spelling errors that could be a source of ambiguity, letters and reports are not edited. Depending on transfer agreements, referee reports obtained elsewhere may or may not be included in this compilation. Referee reports are anonymous unless the Referee chooses to sign their reports.)

Dear Marten,

Thanks again for submitting your Review manuscript (EMBOJ-2026-123587) to The EMBO Journal for our consideration, and for your patience during peer review. Your manuscript has now been seen by two experts in the field, and we have received their very detailed and constructive reports, which you can find appended below.

As you will see, the referees find the Review interesting, relevant, comprehensive, and timely. While they are both largely supportive, they also identify a number of limitations and make several specific suggestions for further improvement, which should help strengthen the Review further and make it a valuable resource for the field.

In light of this input, we would like to invite you to submit a revised version of your Review manuscript taking the detailed comments and suggestions of the referees on board. Please also address all comments in a detailed point-by-point response to the referees' comments, detailing any changes to the manuscript and explaining how the initially raised comments are addressed.

In addition, we kindly request the following editorial points be addressed in the revised manuscript:

- If the Figures contain re-used images or elements of images, including schematics, micrographs or photos, please make sure that you have the permission/license to publish them (this also applies to your own previous work, if the journal you previously published in retains copyright). Certain "creative commons" open access licenses, such as CC-BY 4.0, allow re-use without additional formal permissions. All re-used material must be explicitly cited. If you use an image database for scientific iconography (such as BioRender), please acknowledge it in the respective Figure legends and make sure that you have a license that allows for publication in an academic journal.

- Please remove the Figures from the main manuscript file and only upload them to our online system as individual, high-quality files (as they already are). Their legends should remain in the manuscript, placed below the list of References.

- Please place Table 1 below the Figure legends.

- The Author Contributions statement should be removed from the manuscript file. Instead, we use CRediT to specify the contributions of each author in the journal submission system. Please feel free to use the free text box to provide more detailed descriptions during submission. See also our guide to authors for more information: https://link.springer.com/partners/embo-press/editorial-policies#Authorship.

- The manuscript sections need to be named and ordered as follows: Title page - Abstract - Introduction - Acknowledgements - Disclosure and Competing Interests Statement - References - Figure Legends - Tables.

- Please also note that as part of the EMBO Press transparent editorial process, The EMBO Journal publishes online a Peer Review File along with each accepted manuscript. This File will be published in conjunction with your paper and will include the referee reports, your point-by-point responses and all pertinent correspondence relating to the manuscript. Please let us know in case you want to remove any data or figures from your point-by-point responses before they are published as part of the Peer Review File. Retaining unpublished data in the Peer Review File means that these count as published and that the Peer Review File would need to be referenced in future publications. Please let the editorial office know in case you want to remove any data from this file (contact@embojournal.org).

We look forward to seeing a final version of your manuscript as soon as possible.

Best regards,

Ioannis

Ioannis Papaioannou, PhD
Editor, The EMBO Journal
i.papaioannou@embojournal.org

Referee #1:

Summary

This is a comprehensive and timely review of transcriptional mechanisms governing macrophage identity and activation, with a particular focus on a hierarchical framework involving lineage-determining transcription factors (LDTFs), tissue-restricted transcription factors (TRTFs), and primary and secondary signal-dependent transcription factors (SDTFs). The authors integrate developmental origins, tissue imprinting, cytokine- and TLR-driven activation, transcriptional memory, and disease-associated macrophage phenotypes into a unified conceptual model. The review is well-written, richly referenced, and presents several detailed mechanistic vignettes (e.g. IL 4-STAT6-EGR2-KLF4-PPARγ and IFN γ-STAT1-IRF1) that will be of clear interest to the readership of EMBO Journal.

The main limitations are (i) some redundancy and length that obscure the central message, and (ii) a need to more sharply articulate what is genuinely novel relative to existing reviews on macrophage epigenetics and transcriptional control, especially in the sections on disease and therapeutic targeting. With focused revision to clarify the unique conceptual contribution, tighten some sections, and more explicitly integrate disease-associated macrophage states into the proposed framework, the article could be a valuable resource for the field.

Major points of criticism

Clarify the unique conceptual advance relative to previous macrophage/epigenetic reviews The hierarchical model of LDTFs, tissue-specific TFs, and signal-dependent TFs is compelling and used consistently throughout the review. However, similar frameworks (LDTFs vs stimulus-dependent TFs, enhancer priming, tissue imprinting) have been discussed in earlier reviews, and it is not entirely clear what is fundamentally new here beyond an updated literature synthesis.

Please more explicitly articulate in the Introduction and Conclusion how your concept of "secondary SDTFs" extends existing paradigms. For instance, you might highlight that the review:

formalizes secondary SDTFs as a distinct layer responsible for stimulus-specific chromatin remodeling, late transcriptional responses, and transcriptional memory (e.g. EGR2, PPARγ, IRF1, BATF, MafB);

explicitly integrates this layer with tissue imprinting and developmental ontogeny;

connects TF hierarchy to temporal signal integration, including antagonistic and synergistic interactions (IL 4/IFN γ, IL 4/LPS, IFN γ/LPS, poly(I:C)/CpG, IL 4 + IL 6/IL 10).

Consider adding one short paragraph that explicitly contrasts your framework with prior landmark reviews, explaining what new synthesis or re-interpretation you are providing for readers.

Macrophage plasticity: reconcile apparently conflicting findings and link back to TF/enhancer hierarchy The section on macrophage plasticity nicely summarizes ectopic transfer and ex vivo experiments (Lavin et al., Gosselin et al.) and contrasts these with studies suggesting limited reprogramming potential of mature tissue-resident macrophages (e.g. van de Laar et al.). However, the conceptual reconciliation remains somewhat brief and does not fully exploit your TF/enhancer framework.

Please add a more explicit synthesis that discusses:

how plasticity differs between progenitors/preMacs, monocytes, and fully differentiated TRMs;

the relative importance of ontogeny (EMP vs HSC-derived), niche architecture (e.g. AM vs dermis), and timing of environmental cues;

how stable vs reprogrammable enhancer landscapes and the composition of tissue TF and secondary SDTF networks might mechanistically explain the discrepancies between studies.

It would be helpful if you could explicitly map key plasticity findings onto your hierarchical model (which layer is "locked" versus adjustable in each experimental setting).

Disease-associated macrophages and therapeutic implications: integrate more tightly with the TF hierarchy The sections on rheumatoid arthritis, IBD, lupus nephritis, and other inflammatory diseases provide rich descriptions of disease-associated macrophage (DAM) phenotypes based on scRNA seq, spatial profiling, and functional data. However, the connection back to the central LDTF/TRTF/primary/secondary SDTF framework is not always explicit.

For each major disease context, please more clearly identify:

which TFs act as key "secondary SDTFs" maintaining or reinforcing disease-associated enhancer states (e.g. STAT3, IRF1, IRFs more broadly, NF κB family members);

how these factors relate to the upstream cytokine/PRR environment (e.g. IL 6-STAT3, IFN γ-STAT1/IRF1, type I IFN-ISGF3/IRF family) and to tissue-specific TFs.

When discussing JAK inhibitors and other therapies, please be more explicit about how these interventions perturb the TF hierarchy and associated enhancer landscapes, rather than describing them primarily as reducing "hyperinflammation." For example, can particular disease-associated secondary SDTF modules be inferred to be especially sensitive to JAK inhibition in the cited datasets?

Therapeutic targeting of TF networks: sharpen feasibility and define realistic near-term impact The discussion of TF targeting (e.g. antisense oligonucleotides, cell-type-specific delivery) is interesting but somewhat generic and could be made more concrete and balanced.

Please add one or two specific examples, where available, of attempts to modulate macrophage TFs or their co-factors in vivo (even if not yet clinical) to illustrate feasibility.

At the same time, clarify which aspects you consider realistic in the near term versus clearly speculative. The review could

emphasize that the most immediate translational impact may come from:
using TF and enhancer signatures to stratify patients and monitor therapy response;
targeting upstream signaling nodes (e.g. JAKs) or chromatin modifiers associated with key secondary SDTFs, which may be more druggable than the TFs themselves.
Length, redundancy, and balance across sections the manuscript is lengthy and, at times, somewhat repetitive, which risks obscuring the central conceptual message. The IL 4 and IFN γ sections are particularly detailed and effective, whereas some other parts (e.g. type I IFNs, IL 10/IL 6) are treated more briefly.
Identify and reduce repeated textbook-level explanations of canonical pathways (JAK-STAT, NF κB, basic IFN signaling), focusing instead on their macrophage- and enhancer-specific features.
Where you revisit the same TFs or motifs (e.g. IRF1, ISREs, latent vs de novo enhancers) across different sections, you might replace repeated mechanistic descriptions with explicit cross-references ("as discussed above in the IFN section").
It would also be helpful to explicitly state where key knowledge gaps remain (e.g. IL 10-specific secondary SDTFs, detailed STAT3 chromatin dynamics in macrophages), to turn some of the asymmetry in depth into clearly defined open questions.

Please comment on the suitability of BMDMs to identify in vivo relevant pathways and mechanisms.

Minor points of criticism
Definition and consistent use of "secondary SDTFs"
Text box 3 provides a clear definition of primary vs secondary SDTFs; please ensure this terminology is introduced early in the main text (e.g. Introduction) and applied consistently to the key factors you discuss (EGR2, PPARγ, KLF4, IRF1, BATF, MafB, etc.).
In a few places, "secondary SDTFs" and more generic "downstream TFs" appear to be used interchangeably; tightening this would help readers follow the logic.
Human vs mouse data
Much of the mechanistic work relies on mouse BMDMs and in vivo mouse models, while the disease sections include human single-cell datasets. It would be useful to explicitly indicate where major conclusions are based solely on murine data and where human validation exists, especially in the context of disease-associated states.
Figures and tables
Figure 1 and Table 1 are excellent summaries of tissue-specific cues and TF modules for TRMs. You might consider expanding Table 1 or adding a complementary table that lists key examples of primary vs secondary SDTFs for the main cytokine and TLR stimuli discussed (IL 4, IFN γ, type I IFN, IL 10/IL 6, TLR ligands), along with their main functional roles (early response, memory, antagonism, etc.).
Consider adding an integrative schematic summarizing how homeostatic, acutely stimulated, memory, and disease-associated enhancer landscapes relate to the TF hierarchy and to therapeutic interventions.
Scope and boundaries
Given the breadth of the review, a brief statement clarifying what is intentionally not covered in depth (e.g. non-coding RNAs, detailed metabolic TF networks beyond what is already included) could pre-empt criticisms about omissions and help readers understand the intended scope.
Minor textual issues
I did not systematically check language, but the manuscript is generally well written. A careful proofread for minor typographical inconsistencies and a few long, dense sentences (particularly in the disease sections) would improve readability.
Overall, this is a high-quality and authoritative review that synthesizes a large body of literature into a coherent hierarchical framework for macrophage transcriptional regulation. With revisions to sharpen the conceptual novelty, more tightly integrate disease states and therapeutic perspectives into the TF hierarchy, and streamline some redundancies, the manuscript should be suitable for publication.

Referee #2:

In their review, Drakaki et al focus on transcriptional responses in macrophages as the drivers of their phenotypic and functional states, and potentially new targets in disease. The authors provide a great overview of the multi-level transcriptional and epigenetic regulation that defines macrophage identity throughout development. The review nicely balances the view that macrophage function is determined early in development from their hematopoietic origins vs. the concept that macrophages are plastic and rely on the diverse cues they receive in various tissue environments over life span. The defining of the roles of different transcription factors at multiple stages from early development (LDTFs) through tissue seeding (TRTFs) and functional responses to environmental cues (primary and secondary SDTFs) is helpful for untangling the transcriptional logic that guides macrophage development and function. The summary of the ontogeny and factors that program specific tissue-resident macrophage types is thorough.
There are, however, a few sections that would benefit from restructuring to improve clarity. Also, several omissions should probably be corrected given the comprehensive nature of this review.
1. The nomenclature used to discuss type I and II cytokines is misleading. Historically and broadly, type I (TNF, IFNg) vs type II (IL4, IL13, IL10) cytokines have been viewed as parallel to T-cell responses and represent M1/inflammatory and M2/homeostatic or tissue reparative responses, respectively. These are linked to the downstream physiological outputs rather than types of

receptors, which are somewhat more esoteric and less accessible to general audience. Moreover, it ends up lumping IL10, the key anti-inflammatory type II cytokine, with IFNg, the quintessential inflammatory type I cytokine, into the same group. Since the discussion focuses on the shared downstream events and transcriptional regulation, it makes more sense to adhere to a traditional classification.
2. The authors need to introduce a foundational study that first described the spectral model of macrophage activation in 2014 (PMID: 24530056) and helped bridge the older M1/M2 classification with a modern understanding of the transcriptomic diversity of macrophage states.
3. The review also leaves out a major signal from the glucocorticoid hormones (GCs) which intercepts that from IL4 at multiple levels including critical transcription factor and cofactor interactions (PMID: 37080971, PMID: 22753499, and more). This is important as not only GCs were one of the original 'M2' drivers in the older simplified classification, but GC-polarized macrophages ended up transcriptomically 'closest' to IL4-polarized/ activated macrophages in the unbiased spectral model above.
4. Rheumatoid Arthritis indeed is the prime example of a disease for which there is a potential to perhaps target specific transcription factors. The corresponding section, however, omits enormous amount of groundbreaking single-cell data that emerged from the AMP-RA/SLE Network (PMID: 37938773, PMID: 40091833, PMID: 38821936, PMID: 33879239).
Minor points:
• The abbreviation 'HSC' is introduced twice to refer to two entirely different cell types (Hematopoietic Stem Cells and Hepatic Stellate Cells). Hepatic cells are discussed in a single paragraph and perhaps to do not require an abbreviation at all.
• In the section titled "Transcription factor specification in response to tissue-derived signals", reference is made to timelines of ontogeny of macrophage precursors without mentioning the species.
• In Fig. 1, it may be worth mentioning that CCR2 is required for homing of monocytes to the gut lamina propria (PMID: 25151491), despite the fact that specific tissue signals are not fully understood.

Referee #1:

Summary

This is a comprehensive and timely review of transcriptional mechanisms governing macrophage identity and activation, with a particular focus on a hierarchical framework involving lineage-determining transcription factors (LDTFs), tissue-restricted transcription factors (TRTFs), and primary and secondary signal-dependent transcription factors (SDTFs). The authors integrate developmental origins, tissue imprinting, cytokine- and TLR-driven activation, transcriptional memory, and disease-associated macrophage phenotypes into a unified conceptual model. The review is well-written, richly referenced, and presents several detailed mechanistic vignettes (e.g. IL 4-STAT6-EGR2-KLF4-PPARγ and IFN γ-STAT1-IRF1) that will be of clear interest to the readership of EMBO Journal.

We would like to thank the referee for positively evaluating our review and hope to answer all points of criticism to the best of our abilities.

The main limitations are (i) some redundancy and length that obscure the central message, and (ii) a need to more sharply articulate what is genuinely novel relative to existing reviews on macrophage epigenetics and transcriptional control, especially in the sections on disease and therapeutic targeting. With focused revision to clarify the unique conceptual contribution, tighten some sections, and more explicitly integrate disease-associated macrophage states into the proposed framework, the article could be a valuable resource for the field.

Major points of criticism

Clarify the unique conceptual advance relative to previous macrophage/epigenetic reviews

The hierarchical model of LDTFs, tissue-specific TFs, and signal-dependent TFs is compelling and used consistently throughout the review. However, similar frameworks (LDTFs vs stimulus-dependent TFs, enhancer priming, tissue imprinting) have been discussed in earlier reviews, and it is not entirely clear what is fundamentally new here beyond an updated literature synthesis.

Please more explicitly articulate in the Introduction and Conclusion how your concept of "secondary SDTFs" extends existing paradigms. For instance, you might highlight that the review:

- formalizes secondary SDTFs as a distinct layer responsible for stimulus-specific chromatin remodeling, late transcriptional responses, and transcriptional memory (e.g. EGR2, PPARγ, IRF1, BATF, MafB);
- explicitly integrates this layer with tissue imprinting and developmental ontogeny;
- connects TF hierarchy to temporal signal integration, including antagonistic and synergistic interactions (IL 4/IFN γ, IL 4/LPS, IFN γ/LPS, poly(I:C)/CpG, IL 4 + IL 6/IL 10).

Consider adding one short paragraph that explicitly contrasts your framework with prior landmark reviews, explaining what new synthesis or re-interpretation you are providing for readers.

We thank the reviewer for these comments and acknowledge the lack of clarification on what we consider unique about our conceptualization of the TF hierarchical model. We

would like to emphasize that the novelty of our proposed model lies within the following arguments:

- Seperation of signal-dependent responses in two temporally distinct phases; the first is rapid, somewhat broad, and sometimes (partially) shared between different signaling modalities, whereas the second encodes the specificity and magnitude of the response to the received signal.
- Placement of signal-dependent responses, that have mostly been studied in vitro, within the context of tissue imprinting, ontogeny, and the TF layers that govern resident and recruited macrophage specification.
- Coupling secondary SDTFs to sequential and concurrent integration of distinct signals
- Recounting of how aberrant signaling in inflammatory diseases hijacks the TF hierarchy and drives pathogenic macrophage states, driven by ectopically-induced SDTFs

We have updated the Introduction (Page 4; Lines 84-96) to clearly denote these points and hope they are made clear to the reader. We believe the Conclusion also recounts all of these points.

Rather than contrasting our framework with previous landmark reviews, we consider it a conceptual extension that builds on these foundational studies by integrating recent advances and our conceptual ideas into an updated hierarchical model of macrophage transcriptional regulation.

Macrophage plasticity: reconcile apparently conflicting findings and link back to TF/enhancer hierarchy. The section on macrophage plasticity nicely summarizes ectopic transfer and ex vivo experiments (Lavin et al., Gosselin et al.) and contrasts these with studies suggesting limited reprogramming potential of mature tissue-resident macrophages (e.g. van de Laar et al.). However, the conceptual reconciliation remains somewhat brief and does not fully exploit your TF/enhancer framework.
Please add a more explicit synthesis that discusses:
- how plasticity differs between progenitors/preMacs, monocytes, and fully differentiated TRMs;
- the relative importance of ontogeny (EMP vs HSC-derived), niche architecture (e.g. AM vs dermis), and timing of environmental cues;
- how stable vs reprogrammable enhancer landscapes and the composition of tissue TF and secondary SDTF networks might mechanistically explain the discrepancies between studies.

It would be helpful if you could explicitly map key plasticity findings onto your hierarchical model (which layer is "locked" versus adjustable in each experimental setting).

We agree with the reviewer that the section on Macrophage Plasticity previously lacked a reconciliation of the conflicting findings presented on one side by Lavin et al., claiming that mature TRMs display extensive plasticity, and on the other side by van de Laar et al., questioning that paradigm. Additionally, we had not explicitly stated our position on this "debate", or tried to utilize our hierarchical TF model to explain the discrepancies between these studies.

We believe that the conflicting findings can be explained, to a large extent, by the different models used to address the question of plasticity in these two studies. Lavin and colleagues transferred matured peritoneal macrophages into an intact alveolar macrophage niche. Recovered cells, isolated 15 days following transfer, represented a very small fraction of the total alveolar macrophage population. Naturally, chromatin dynamics were not assessed in this small sample size, neither was their ability to perform alveolar macrophage functions. The study also left an unanswered question - would these cells succesfully engraft and represent a considerable proportion of the population given more time in the niche? In the paper of van de Laar et al., *Csf2rb-/-* mice serve as an elegant model to address the question of the extent of macrophage plasticity. Fully differentiated macrophages from three different tissues, including the peritoneum, were transferred into the empty alveolar macrophage niche of these mice, and isolated again after 6 weeks. In this context, no mature macrophage population could efficiently engraft the empty niche or develop into a functional alveolar macrophage phenotype. In contrast, different types of precursor cells, including yolk sac- and fetal-derived precursors and bone-marrow derived monocytes maintained enough plasticity to adopt an alveolar macrophage identity.

We think the findings by van de Laar and colleagues most faithfully represent the reality of macrophage plasticity. We extend this idea by drawing on observations from other groups showing that tissue perturbation drives the recruitment of monocytes that differentiate into specialized macrophage subsets, participating in the distinct phases of the inflammatory response and its resolution. Under these conditions, resident macrophage numbers are reduced due to tissue damage, shifting their priority toward self-renewal and maintenance of homeostatic functions rather than active participation in the damage response.

We have updated this whole section and hope to have provided reconciliation of the contrasting findings through this literature exploration. We have also tried to provide more detailed commentary on how plasticity differs between different types of precursor cells and mature resident macrophages, and how reversible or stable enhancer landscapes may explain these differences in light of the discussed studies.

Disease-associated macrophages and therapeutic implications: integrate more tightly with the TF hierarchy The sections on rheumatoid arthritis, IBD, lupus nephritis, and other inflammatory diseases provide rich descriptions of disease-associated macrophage (DAM) phenotypes based on scRNA seq, spatial profiling, and functional data. However, the connection back to the central LDTF/TRTF/primary/secondary SDTF framework is not always explicit.
For each major disease context, please more clearly identify:
which TFs act as key "secondary SDTFs" maintaining or reinforcing disease-associated enhancer states (e.g. STAT3, IRF1, IRFs more broadly, NF κB family members);
how these factors relate to the upstream cytokine/PRR environment (e.g. IL 6-STAT3, IFN γ-STAT1/IRF1, type I IFN-ISGF3/IRF family) and to tissue-specific TFs.

We thank the reviewer for this helpful suggestion. We agree that the connection between disease-associated macrophage states and the TF hierachy is not always as

explicit. It is difficult to define specific secondary SDTFs in chronic disease settings because of the complex mixed (cytokine-) environment. We have added a paragraph explaining that, in chronic inflammatory settings, the distinction between primary and secondary SDTFs becomes less clear. We therefore refer to these collectively as disease-associated SDTFs, which act together with lineage-defining and tissue-resident TFs to maintain pathogenic macrophage identities (p. 23, lines 752-758).

In addition, we revised the RA, IBD, and lupus sections to more clearly link the inflammatory environment with the disease-associated SDTF networks likely involved in each context (p. 24, lines 776-785) (p. 25 , 846-852) (p. 26, 867-870).

When discussing JAK inhibitors and other therapies, please be more explicit about how these interventions perturb the TF hierarchy and associated enhancer landscapes, rather than describing them primarily as reducing "hyperinflammation." For example, can particular disease-associated secondary SDTF modules be inferred to be especially sensitive to JAK inhibition in the cited datasets?

We appreciate the reviewer's comment and acknowledge that perhaps the section on JAK inhibition included in Text box 4 has caused some confusion. To combat this, we have provided an example of how JAK inhibition in rheumatoid arthritis may not only interfere with the pro-inflammatory pathways it is trying to dampen, but also with pathways that promote reparative macrophage polarization, demonstrating the counterproductive consequences of targeting non-specific regulators of signal responses. (Text box 4)

Therapeutic targeting of TF networks: sharpen feasibility and define realistic near-term impact.
The discussion of TF targeting (e.g. antisense oligonucleotides, cell-type-specific delivery) is interesting but somewhat generic and could be made more concrete and balanced.
Please add one or two specific examples, where available, of attempts to modulate macrophage TFs or their co-factors in vivo (even if not yet clinical) to illustrate feasibility.
At the same time, clarify which aspects you consider realistic in the near term versus clearly speculative. The review could emphasize that the most immediate translational impact may come from:
using TF and enhancer signatures to stratify patients and monitor therapy response;
targeting upstream signaling nodes (e.g. JAKs) or chromatin modifiers associated with key secondary SDTFs, which may be more druggable than the TFs themselves.

We appreciate these comments from Reviewer #1 concerning the section on therapeutic modulation of macrophage TFs. We recognize the added value of providing examples of attempts to specifically target macrophage TFs to bring our point across. To this end, we disclose a recent study that identifies the transcription factor ETS2 as a major driver of macrophage inflammatory programs in inflammatory bower disease. Inhibition of MEK, an upstream regulator of ETS2, in intestinal biopsies of IBD reduced inflammatory cytokine production similarly to anti-TNF therapy. The authors of this study also identify the need for ETS2 inhibition, specifically addressed to macrophages, to overcome the toxicities associated with MEK inhibition

(https://doi.org/10.1038/s41586-024-07501-1) (p. 25, Lines 885-894). Additionally, we have included a sentence that clarifies that most research on TF inhibitors and macrophage-specific delivery remain in early stages (p. 26, lines 912-913). We have also highlighted the immediate translation impact of our TF hierarchical model (p. 26, lines 917-922) as the Reviewer suggested.

Length, redundancy, and balance across sections the manuscript is lengthy and, at times, somewhat repetitive, which risks obscuring the central conceptual message. The IL 4 and IFN γ sections are particularly detailed and effective, whereas some other parts (e.g. type I IFNs, IL 10/IL 6) are treated more briefly.
Identify and reduce repeated textbook-level explanations of canonical pathways (JAK-STAT, NF κB, basic IFN signaling), focusing instead on their macrophage- and enhancer-specific features. Where you revisit the same TFs or motifs (e.g. IRF1, ISREs, latent vs de novo enhancers) across different sections, you might replace repeated mechanistic descriptions with explicit cross-references ("as discussed above in the IFN section").
It would also be helpful to explicitly state where key knowledge gaps remain (e.g. IL 10-specific secondary SDTFs, detailed STAT3 chromatin dynamics in macrophages), to turn some of the asymmetry in depth into clearly defined open questions.

We thank the reviewer for this suggestion. We have revised the manuscript to reduce redundancy and condense descriptions of canonical signaling pathways where appropriate, while retaining brief mechanistic overviews that are necessary. We have also highlighted key knowledge gaps, including additional IFN-I-associated SDTFs (p. 14, line 414-416) and IL-10/IL-6-specific secondary SDTFs (p. 17, line 537-540) to clarify areas requiring further investigation

Please comment on the suitability of BMDMs to identify in vivo relevant pathways and mechanisms.

We have added a clarification on the suitability and limitations of BMDMs (p. 13, line 399 - 403)

Minor points of criticism
Definition and consistent use of "secondary SDTFs"
Text box 3 provides a clear definition of primary vs secondary SDTFs; please ensure this terminology is introduced early in the main text (e.g. Introduction) and applied consistently to the key factors you discuss (EGR2, PPARγ, KLF4, IRF1, BATF, MafB, etc.). In a few places, "secondary SDTFs" and more generic "downstream TFs" appear to be used interchangeably; tightening this would help readers follow the logic.

We thank the reviewer for this suggestion. We now introduce the concepts of primary and secondary SDTFs in the Introduction and have revised the manuscript to consistently use this terminology throughout. Where appropriate, we replaced the more generic term “downstream TFs” with “secondary SDTFs” to improve clarity and consistency.

Human vs mouse data

Much of the mechanistic work relies on mouse BMDMs and in vivo mouse models, while the disease sections include human single-cell datasets. It would be useful to explicitly indicate where major conclusions are based solely on murine data and where human validation exists, especially in the context of disease-associated states.

We have clarified throughout the text when findings are derived from mouse models versus human studies.

Figures and tables
Figure 1 and Table 1 are excellent summaries of tissue-specific cues and TF modules for TRMs. You might consider expanding Table 1 or adding a complementary table that lists key examples of primary vs secondary SDTFs for the main cytokine and TLR stimuli discussed (IL 4, IFN γ, type I IFN, IL 10/IL 6, TLR ligands), along with their main functional roles (early response, memory, antagonism, etc.).
Consider adding an integrative schematic summarizing how homeostatic, acutely stimulated, memory, and disease-associated enhancer landscapes relate to the TF hierarchy and to therapeutic interventions.

We thank the reviewer for these suggestions. We considered adding such a table but decided against it because secondary SDTF networks are currently well defined for only some pathways (e.g., IL-4 and IFN-γ), while remaining poorly characterized for others. A comparative table would therefore be asymmetric, and we instead highlight these knowledge gaps throughout the manuscript. We also considered adding an additional integrative schematic. However, we feel that the current figures already capture the central concepts of our proposed hierarchical framework, and that adding disease/homeostatic TFs, memory, interventions and the hierarchy all in one figure will result in a very complex/unreadable figure.

Scope and boundaries
Given the breadth of the review, a brief statement clarifying what is intentionally not covered in depth (e.g. non-coding RNAs, detailed metabolic TF networks beyond what is already included) could pre-empt criticisms about omissions and help readers understand the intended scope.

In our most recent version, we made sure to include our focus and what we could not cover in our review to the Introduction (p.4, lines 81-84).

Minor textual issues
I did not systematically check language, but the manuscript is generally well written. A careful proofread for minor typographical inconsistencies and a few long, dense sentences (particularly in the disease sections) would improve readability.
Overall, this is a high-quality and authoritative review that synthesizes a large body of literature into a coherent hierarchical framework for macrophage transcriptional regulation. With revisions to sharpen the conceptual novelty, more tightly integrate disease states and therapeutic perspectives into the TF hierarchy, and streamline some redundancies, the manuscript should be suitable for publication.

Minor textual issues have been resolved along the way.

Referee #2:

In their review, Drakaki et al focus on transcriptional responses in macrophages as the drivers of their phenotypic and functional states, and potentially new targets in disease. The authors provide a great overview of the multi-level transcriptional and epigenetic regulation that defines macrophage identity throughout development. The review nicely balances the view that macrophage function is determined early in development from their hematopoietic origins vs. the concept that macrophages are plastic and rely on the diverse cues they receive in various tissue environments over life span. The defining of the roles of different transcription factors at multiple stages from early development (LDTFs) through tissue seeding (TRTFs) and functional responses to environmental cues (primary and secondary SDTFs) is helpful for untangling the transcriptional logic that guides macrophage development and function. The summary of the ontogeny and factors that program specific tissue-resident macrophage types is thorough.

There are, however, a few sections that would benefit from restructuring to improve clarity. Also, several omissions should probably be corrected given the comprehensive nature of this review.
1. The nomenclature used to discuss type I and II cytokines is misleading. Historically and broadly, type I (TNF, IFNg) vs type II (IL4, IL13, IL10) cytokines have been viewed as parallel to T-cell responses and represent M1/inflammatory and M2/homeostatic or tissue reparative responses, respectively. These are linked to the downstream physiological outputs rather than types of receptors, which are somewhat more esoteric and less accessible to general audience. Moreover, it ends up lumping IL10, the key anti-inflammatory type II cytokine, with IFNg, the quintessential inflammatory type I cytokine, into the same group. Since the discussion focuses on the shared downstream events and transcriptional regulation, it makes more sense to adhere to a traditional classification.

We have revised the section organization to focus on interferon-mediated and interleukin-mediated transcriptional regulation, avoiding the potentially misleading type I/type II cytokine terminology while better reflecting the downstream transcriptional mechanisms discussed (p.13-16)

2. The authors need to introduce a foundational study that first described the spectral model of macrophage activation in 2014 (PMID: 24530056) and helped bridge the older M1/M2 classification with a modern understanding of the transcriptomic diversity of macrophage states.

Thanks for catching this, we now introduce this model as well as the nomeclature paper by Murray et al. (p.13, line 381-387)

3. The review also leaves out a major signal from the glucocorticoid hormones (GCs) which intercepts that from IL4 at multiple levels including critical transcription factor and cofactor interactions (PMID: 37080971, PMID: 22753499, and more). This is important as not only GCs were one of the original 'M2' drivers in the older simplified

classification, but GC-polarized macrophages ended up transcriptomically 'closest' to IL4-polarized/ activated macrophages in the unbiased spectral model above.

We thank the reviewer for highlighting the importance of glucocorticoid signaling in macrophage regulation. We have added a new section discussing GC-mediated transcriptional regulation. We now disucss its convergence with IL-4/STAT6-driven enhancer programs and its ability to modulate NF-κB-dependent inflammatory responses (p. 21-22, lines 703 - 722)

4. Rheumatoid Arthritis indeed is the prime example of a disease for which there is a potential to perhaps target specific transcription factors. The corresponding section, however, omits enormous amount of groundbreaking single-cell data that emerged from the AMP-RA/SLE Network (PMID: 37938773, PMID: 40091833, PMID: 38821936, PMID: 33879239).

These papers were very good suggestions. We have expanded the RA section to incorporate the relevant single-cell data describing inflammatory and tissue-resident myeloid states and their associated TF regulatory programs (p. 24, lines 784-807)

Minor points:

- The abbreviation 'HSC' is introduced twice to refer to two entirely different cell types (Hematopoietic Stem Cells and Hepatic Stellate Cells). Hepatic cells are discussed in a single paragraph and perhaps to do not require an abbreviation at all.

We are grateful to the reviewer for this observation. We have removed the abbreviation for hepatic stellate cells in the section 'Liver Kupffer cells' (p. 8, line 259).

- In the section titled "Transcription factor specification in response to tissue-derived signals", reference is made to timelines of ontogeny of macrophage precursors without mentioning the species.

We thank the reviewer for identifying this oversight. We now clarify that the observations discussion in the section "Transcription factor specification in response to tissue-derived signals" are based on mouse studies (p. 5, Line 106).

- In Fig. 1, it may be worth mentioning that CCR2 is required for homing of monocytes to the gut lamina propria (PMID: 25151491), despite the fact that specific tissue signals are not fully understood.

We appreciate this comment and agree that monocyte recruitment to the gut lamina propria, as well as to tissues in response to damage is CCR2-dependent. However, this axis is not discussed anywhere in the main text, as we felt it might be out of scope for this review. Therefore, including it in the figure might be confusing for the readers.

2nd Revision - Editorial Decision **23rd Jul 2026**

Dear Marten,

Congratulations on an excellent manuscript! I am very pleased to inform you that your Review article has been accepted for publication in The EMBO Journal. Thank you for comprehensively addressing the referees' comments and concerns, as well as our editorial requests for changes and corrections.

Your manuscript will be processed for publication by EMBO Press. It will be copy edited and you will receive page proofs prior to publication. Please note that you will be contacted by Springer Nature Author Services to complete licensing information.

Should you be planning a Press Release on your article, please notify the production team via this form in order to coordinate publication and release dates: https://forms.cloud.microsoft/e/eH4A8JVP6g

If you have any questions, please do not hesitate to contact the Editorial Office. Thank you for your contribution to The EMBO Journal. Working with you has been a pleasure.

Best regards,

Ioannis

Ioannis Papaioannou, PhD
Senior Editor, The EMBO Journal - EMBO Reports - Molecular Systems Biology
i.papaioannou@embojournal.org

*****

Please note that it is The EMBO Journal policy for the transcript of the editorial process (containing referee reports and your response letters) to be published as an online supplement to each paper. If you should prefer removal of any referee-only figures included in the point-by-point response(s), e.g. because they may still be used for future publication or because they have been reproduced from published work by others, please do let us know immediately via response email.
More information is available here: https://link.springer.com/partners/embo-press/editorial-policies#Peer%20review
